# Metabolite profiling of barley flag leaves under drought and combined heat and drought stress reveals metabolic QTLs for metabolites associated with antioxidant defense

**DOI:** 10.1093/jxb/erx038

**Published:** 2017-03-10

**Authors:** Sven Eduard Templer, Alexandra Ammon, David Pscheidt, Otilia Ciobotea, Christian Schuy, Christopher McCollum, Uwe Sonnewald, Anja Hanemann, Jutta Förster, Frank Ordon, Maria von Korff, Lars Matthias Voll

**Affiliations:** 1 Julius Kühn-Institute, Federal Research Centre for Cultivated Plants, Institute of Resistance Research and Stress Tolerance, Erwin-Baur-Str. 27, D-06484 Quedlinburg, Germany; 2 Max Planck Institute for Breeding Research, Carl-von-Linné-Weg 10, D-50829 Köln, Germany; 3 Friedrich-Alexander-Universität Erlangen-Nürnberg, Division of Biochemistry, Staudtstr. 5, D-91058 Erlangen, Germany; 4 Saatzucht Josef Breun GmbH & Co. KG, Amselweg 1, D-91074 Herzogenaurach, Germany; 5 SAATEN-UNION BIOTEC GmbH, Hovedisser Strasse 92, D-33818 Leopoldshöhe, Germany; 6 Cluster of Excellence on Plant Sciences, Heinrich-Heine-Universität Düsseldorf, Institute for Plant Genetics, Universitätsstrasse 1, D-40225 Düsseldorf, Germany

**Keywords:** Barley, combined heat and drought stress, GABA shunt, genome-wide association mapping, glutathione, metabolite profiling, mQTL, thousand grain weight, tocopherol.

## Abstract

Barley (*Hordeum vulgare* L.) is among the most stress-tolerant crops; however, not much is known about the genetic and environmental control of metabolic adaptation of barley to abiotic stresses. We have subjected a genetically diverse set of 81 barley accessions, consisting of Mediterranean landrace genotypes and German elite breeding lines, to drought and combined heat and drought stress at anthesis. Our aim was to (i) investigate potential differences in morphological, physiological, and metabolic adaptation to the two stress scenarios between the Mediterranean and German barley genotypes and (ii) identify metabolic quantitative trait loci (mQTLs). To this end, we have genotyped the investigated barley lines with an Illumina iSelect 9K array and analyzed a set of 57 metabolites from the primary C and N as well as antioxidant metabolism in flag leaves under control and stress conditions. We found that drought-adapted genotypes attenuate leaf carbon metabolism much more strongly than elite lines during drought stress adaptation. Furthermore, we identified mQTLs for flag leaf γ-tocopherol, glutathione, and succinate content by association genetics that co-localize with genes encoding enzymes of the pathways producing these antioxidant metabolites. Our results provide the molecular basis for breeding barley cultivars with improved abiotic stress tolerance.

## Highlight

mQTLs for barley flag leaf γ-tocopherol, glutathione, and succinate content co-localize with genes encoding enzymes of the pathways producing these antioxidant metabolites.

## Introduction

Heat and drought severely limit crop productivity worldwide and often prevail in combination. Prolonged heat and/or drought periods are not limited to arid and semi-arid regions, but also increasingly affect European agriculture in the course of global climate change. Based on regional and global models, a progressive redistribution of precipitation to winter months and increased temperatures in spring and summer are predicted for Central Europe (e.g. Spekat *et al.*, 2007; Estrella and Menzel, 2013). Central European cultivation areas are already increasingly subject to intermittent and terminal drought and heat stress, which generally have the strongest effects on yield during the reproductive phase of barley (Jamieson *et al.*, 1995; Barnabás *et al.*, 2008; Dolferus *et al.*, 2011).

Barley (*Hordeum vulgare* L.) breeding in temperate regions such as Central Europe was conducted under favorable environmental conditions with a focus on yield improvement rather than abiotic stress tolerance. In contrast, the wild and cultivated progenitors of Central European breeding lines from the Fertile Crescent are well adapted to heat and drought stress. Several independent approaches have demonstrated that wild barley (*H. vulgare* spp. *spontaneum*) as well as barley landraces from the Mediterranean area are valuable genetic resources that can be exploited to improve drought and/or heat tolerance in elite germplasm (Teulat *et al.*, 2001*b*, ; Talame *et al.*, 2004; von Korff *et al.*, 2004, 2006; Ceccarelli *et al.*, 2007; Worch *et al.*, 2011; Kalladan *et al.*, 2013, Rollins *et al.*, 2013; Mansour *et al.*, 2014; Tondelli *et al.*, 2014; Mikolajczak *et al.*, 2016). Drought and heat tolerance are complex genetic traits governed by multiple genes that affect several developmental, morphological, and physiological processes. From an agronomic point of view, drought and/or heat tolerance ultimately result in yield stability (von Korff *et al.*, 2006; Worch *et al.*, 2011; Tondelli *et al.*, 2014; Mikołajczak *et al.*, 2016), which can be achieved by three different strategies: escape, avoidance, or tolerance. Escape strategists prevent excessive yield loss by stress-triggered reprogramming of reproductive development, ultimately leading to premature flowering (for a recent study, see Obsa *et al.*, 2016). Avoidance comprises physiological responses that improve water use efficiency, such as stomatal closure (e.g. Teulat *et al.*, 2002, 2003). In contrast, stress tolerance is caused by improved cellular adaptation to the stress situation (as reviewed by Krasensky and Jonak, 2012). Quantitative trait loci (QTLs) studies have identified genomic regions controlling agronomic performance under water-limiting conditions in the field and under control conditions (Baum *et al.* 2003; Teulat *et al.*, 2003; von Korff *et al.*, 2006; Worch *et al.*, 2011; Kalladan *et al.*, 2013; Tondelli *et al.*, 2014; Wehner *et al.*, 2015, 2016; Mikolajczak *et al.*, 2016). These studies have primarily explored the genetic control of morphological and physiological adaptation to stress in barley. However, abiotic stresses are known to have profound effects on plant metabolism. The accumulation of compatible solutes such as sugars, proline, fructans, glycine betaine, and polyamines is associated with increased drought tolerance in plants (for a recent review, see Krasensky and Jonak, 2012). QTLs for proline and soluble sugar accumulation as well as leaf osmotic potential have been identified in drought-stressed barley (Teulat *et al.*, 2001*a*, 2003; Sayed *et al.*, 2012). However, a comprehensive survey of the genetic variation of metabolic adaptation to heat and drought is lacking for barley. Understanding the genetic differences in metabolic adaptation of barley is therefore crucial to breed barley varieties that are well prepared to experience abiotic stresses.

The recent development of robust metabolomics protocols and the establishment of metabolomics platforms have enabled the investigation of metabolic QTLs (mQTLs), which can be explored in a similar fashion to QTLs for agronomic and morphological traits. To detect an mQTL, the genome-wide association between the quantitative accumulation of a metabolite of interest and alleles of molecular markers is calculated from a large set of genotypes, revealing the genetic basis for metabolic variation (Schauer *et al.*, 2006; Carreno-Quintero *et al.*, 2012). Since the discipline is quite young, only a few reports exist on the identification of mQTLs and their correlation with morphological changes in response to abiotic stresses in crop plants (Schauer *et al.*, 2006; Carreno-Quintero *et al.*, 2012; Riedelsheimer *et al.*, 2012; Wen *et al.*, 2015). Using maize recombinant inbred lines, Wen *et al.* (2015, 2016) identified ~50 mQTLs for major compounds from carbon and nitrogen metabolism that co-localized with 34 candidate genes of the corresponding pathways. By computing metabolite–metabolite–agronomic trait networks, all scored agronomic traits could partly be explained by metabolite levels (Wen *et al.*, 2015). Using a genome-wide association approach with 289 maize inbred lines, Riedelsheimer *et al.* (2012) were able to detect 15 significant marker–metabolite associations and identified correlations between metabolites and morphological traits. A recent study in wheat revealed 95 mQTLs in drought-stressed flag leaves, 38 of which shared their position with QTLs for agronomic traits under terminal drought stress (Hill *et al.*, 2013). These studies suggest that metabolite levels represent promising connecting links for narrowing the genotype–phenotype gap of complex agronomic traits.

We hypothesized that differences in stress tolerance between the elite and landrace barley genotypes could in part be caused by variation in metabolic adaptation to heat and drought stress. Consequently, our objectives were (i) to investigate potential differences in the metabolic adaptation of barley flag leaves to terminal drought and combined heat and drought stress and (ii) to identify mQTLs.

## Materials and methods

### Plant material and growth conditions

A genetically diverse set of 81 two-rowed spring barley genotypes, comprising 35 landrace and elite genotypes adapted to semi-arid areas from all over the world (termed Mediterranean/MED hereafter) as well as 46 elite German breeding lines and cultivars adapted to the Central European climate (termed GER hereafter), was subjected to controlled drought and combined heat and drought stress in climate chamber experiments (see Supplementary Table S1 at *JXB* online). Four plants per genotype were grown per pot in 4.5 liters of Einheitserde ED73 (Gebr. Patzer, Sinntal, Germany) in 16 h (20 °C) light/8 h (16 °C) dark cycles until BBCH growth stage 49 (Lancashire *et al.*, 1991), at which time one pot per genotype was subjected to control, drought, and combined drought and heat conditions, respectively. Three independent experiments each with four plants in one pot per treatment were analyzed.

To synchronize flowering time, genotypes of Mediterranean origin were grown in short-day conditions (8 h light/16 h dark) for the first 4 weeks (as indicated in Supplementary Table S1), and were then transferred to long-day conditions. In addition, a preliminary experiment was used to estimate time to heading under controlled chamber conditions and to separate genotypes into three groups sown on days 0, 7, and 14. The relative humidity of the chambers was set to 50%, the light intensity was 20 klux and the temperature was set to 20 °C light/16 °C dark. The field capacity (FC) was calculated as the difference in weight between fully hydrated soil and dried soil (Colman, 1947). The soil water content (SWC) of potted plants was adjusted to 70% of the FC. Drought conditions were established by withholding water until the SWC was reduced to 30% FC, which was reached after 3 d The SWC was maintained for 14 d until plant maturity, when watering was stopped. For the combined heat and drought treatment, the temperature was increased from 20 °C/16 °C during day/night under control conditions to 26 °C/20 °C at the time 30% FC was reached until harvest.

### Plant phenotyping

During the stress treatment, leaf temperature was determined with an Optris LaserSight infrared thermometer (Optris GmbH, Berlin, Germany) and the quantum yield of photosynthesis was recorded using an OS1p device (ADC BioScientific Ltd, Herts, UK). Measurements were performed in the middle of the light period on the last leaf before the flag leaf at days 1 and 7 after a FC of 30% had been reached. At day 8, an ~3 cm long segment of the same leaf was sampled to determine the relative water content (RWC) according to the equation: $\frac{\text{freshweight}–\text{dryweight}}{\text{turgidweight}–\text{dryweight}}\times 100$. Leaf segments were immediately weighed to determine the fresh weight. The turgid weight was determined after submerging the cuttings in distilled water and storing overnight at 4 °C in the dark. Dry weight was determined after drying the cuttings at 95 °C for 24 h. At harvest, agronomic traits were recorded: plant height (HGT) was measured from the lowest node to the top of the ear of the largest tiller. Straw biomass (YST), grain weight (YGR), kernel number per ear (KPE), as well as thousand kernel weight (TKW) were recorded after drying plant material at 95 °C for 24 h.

### Leaf sampling for metabolite profiling

Flag leaves of barley plants grown under control, drought, and combined drought and heat stress were sampled in the middle of the light period into four pools of five leaves each at day 3 after an FC of 30% was established, and were used for metabolite analysis. The leaf samples were analyzed for 57 metabolites, major antioxidants, soluble sugars, amino acids, carboxylates, and phosphorylated intermediates as described below.

#### Quantification of tocopherol, soluble sugars, starch, and free amino acids

Tocopherol, soluble sugar, starch, and free amino acid contents were determined from aliquots of 20–30 mg of leaf tissue as described in Abbasi *et al.* (2007). While tocopherol and amino acid contents were determined after HPLC separation by fluorescence detection, soluble sugars and starch were quantified using a spectrophotometric assay.

#### Determination of glutathione levels

The contents of oxidized and reduced glutathione were determined from 30 mg of leaf tissue aliquots by reversed-phase HPLC following the protocol of Abbasi *et al.* (2009).

#### Quantification of intermediates of major carbohydrate and carboxylate metabolism

Phosphorylated intermediates and major carboxylic acids were determined by ion chromatography–tandem MS (IC-MS/MS) of perchloric acid extracts of 50–100 mg of leaf tissue as described by Horst *et al.* (2010).

### Plant genotyping

All barley genotypes investigated in this study were genotyped by Traitgenetics GmbH (Gatersleben, Germany) with an Illumina iSelect 9K SNP array containing 7864 genome-wide single nucleotide polymorphism (SNP) markers (Comadran *et al.*, 2011). To this end, genomic DNA was extracted from 50 mg of leaf material from young seedlings using the Biosprint 96 DNA Plant Kit (Qiagen GmbH, Hilden, Germany). The SNP array contains 3967 markers that are mapped (Close *et al.*, 2009). Of these, 2596 SNPs were selected that had a minor allele frequency of at least 10% and maximum missing data frequency of 10% in the total population of genotypes.

### Statistical analysis

All statistical analyses were performed using the statistical language R (R Core Team, 2016). The squared correlation *r*^2^ of marker pairs was calculated to obtain linkage disequilibrium (LD) values as in Flint-Garcia *et al.* (2003). To average the LD extent over the genetic distance, a linear regression was calculated according to Hill and Weir (1988). The 95% quantile of the distribution of LD of interchromosomal pairs was used as background LD. The intersection of LD regression and background was set as average genome-wide LD decay.

In order to calculate phenotypic values of agronomic and physiological performance within each treatment, the best linear unbiased estimates (BLUES) for each genotype were estimated as in the equation:

$${\text{y}}_{\text{ijk}}={\text{g}}_{\text{j}}+{\text{r}}_{\text{k}}+{\text{p}}_{\text{ijk}}+{\text{q}}_{\text{ijk}}+{\text{c}}_{\text{ijk}}+\text{e}$$

where y_ijk_ is the phenotypic value of the *i*th plant of the *j*th genotype in the *k*th experimental repeat; r is the random effect of the experimental repeat, p, q, and c are the random effects of pot positions by row (p) and column (q) within the chamber c. e is the unknown random error.

BLUES were used for correlation analysis and marker trait associations as described below.

Pearson correlation coefficients were calculated between all phenotypic and metabolic traits for each treatment separately. Marker trait associations were calculated with a mixed linear model as in the equation:

$${\text{y}}_{\text{jk}}={\text{s}}_{\text{i}}+{\text{t}}_{\text{j}}+{\text{g}}_{\text{k}}+\text{e}$$

with y_jk_ as the phenotypic BLUE of the *i*th genotype under *k*th treatment, while s is the fixed effect of the *i*th SNP and t the fixed effect of the *j*th treatment. g is the random effect of genotype k from the additive relationship matrix according to Endelmann and Jannink (2012), and e is the random unknown error.

 The false discovery rate (FDR) was set to 5% for calculating the probability threshold.

Phenotypic variations among genotypes and environments were visualized by boxplots for the GER and the MED germplasm sets separately. In addition, a principle component analysis (PCA) was performed for all centered and scaled (by SD) phenotypic and metabolic values using singular value decomposition (Venables and Ripley, 2002). Scores and loadings were plotted in a biplot as defined by Gabriel (1971). Significance of effects of the treatment, the germplasm group, and their interaction was calculated with a two-factorial ANOVA according to the model:

$$\text{yij}={\text{t}}_{\text{j}}+{\text{g}}_{\text{k}}+{\text{t}}_{\text{j}}\times {\text{g}}_{\text{k}}+\text{e}$$

where y is the phenotypic BLUE of the *i*th genotype under *j*th treatment, t is the fixed effect of the treatment, and g the fixed effect of the *k*th germplasm group classified for the *i*th genotype.

## Results

The aim of this study was to identify genetic differences in the adaptation of barley flag leaf metabolism to drought and combined heat and drought stress. Furthermore, we were interested in identifying flag leaf metabolite markers in soluble carbon, nitrogen, and antioxidant metabolism that correlate with plant performance under drought or combined heat and drought stress. To this end, a genetically diverse set of 81 barley lines, comprising 35 diverse landraces and cultivars adapted to semi-arid areas and 46 German elite breeding lines (see Supplmentary Table S1) were compared for phenotypic and metabolic differences under drought and combined heat and drought stress applied at anthesis. Using genotyping data of the 81 investigated barley accessions and metabolite data from three different environmental conditions, our final aim was to reveal mQTLs.

### German breeding lines are genetically distinct from the diverse barley collection

**Fig. 1.**
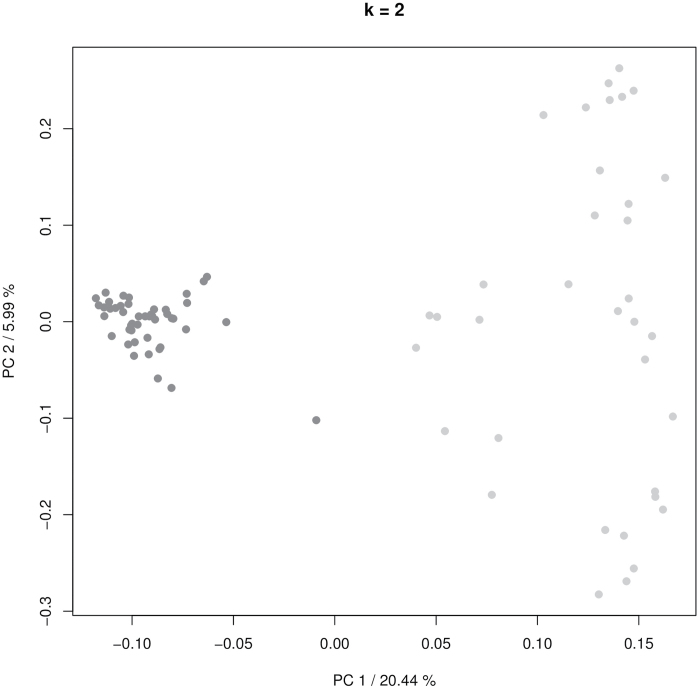
Genetic structure of the investigated barley collection. The genetic diversity of the investigated barley panel was analysed by k-means clustering (*k*=2) of 2596 informative SNP markers of the Illumina 9K SNP array from all 81 investigated genotypes. German breeding lines are represented by dark grey dots, while the diverse barley accessions are shown as light grey dots.

We first evaluated the genetic structure and LD in the diverse germplasm set. To this end, we used 2596 of the 3967 mapped polymorphic SNP markers of the Illumina 9K SNP array that had a minor allele frequency of at least 10% and a maximum missing data frequency of 10% in the entire population. The average LD decay in the population was determined at 3.5 cM based on the *r*^2^ between all intrachromosomal pairs of loci (Supplementary Fig. S1). K-means clustering (*k*=2; Fig. 1) and PCA (Supplementary Fig. S2) based on the polymorphic mapped 2596 SNPs clearly separated the German breeding lines from the diverse landrace and cultivated genotypes (Fig. 1; Supplementary Fig. S2). Consequently, the germplasm set showed a strong classification in two genetic clusters which separated the German breeding lines and cultivars from the landrace and cultivar genotypes originating from Mediterranean environments. To evaluate if the genetic differences were also reflected in a clear phenotypic separation, we analyzed morphological, physiological, and metabolic performance for the two germplasm sets separately.

### Grain weight is more stable in drought-adapted accessions

We first evaluated genetic and stress-induced differences in physiological and morphological traits. For this purpose, we compared the impact of the three different treatments, control, drought, and drought/heat on the physiological response and morphological traits of the GER and MED subpanels.

**Fig. 2.**
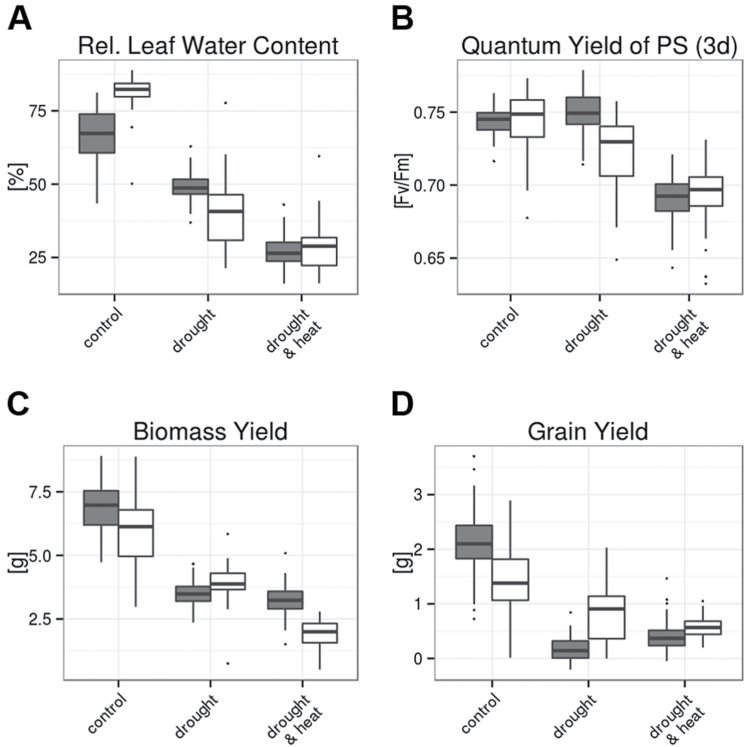
Physiological status in the early stress phase and morphological traits of GER and MED lines at terminal harvest. (A) Relative leaf water content (RWC) at 8 d after stress establishment in %. (B) *F*_v_/*F*_m_ value (maximum quantum yield of PSII, i.e. PSII intactness) on the first day after stress establishment. (C) Total biomass (straw plus grain yield) at terminal harvest in g DW. (D) Grain yield at terminal harvest in g DW. Data of four replicates per genotype and treatment from 46 GER lines (gray bars) and 35 MED lines (white bars) were grouped for analysis and are shown from control (left), drought stress (middle), and combined heat and drought (right). In the box plots, the median± upper and lower quartile are indicated as boxes, while whiskers indicate values within 1.5 times the interquartile range away from median from the lower and upper quartile. Dots reflect outliers of these regions (Tukey, 1977). Results of the ANOVA are provided in Supplementary Table S3. (This figure is available in colour at *JXB* online.)

After a prolonged period of 8 d after stress establishment, drought and combined stresses led to a reduction in relative water content (RWC) in the leaf below the flag leaf from >70% in control conditions to ~50% and 30% under drought and combined stresses, respectively (Fig. 2A). In drought stress, RWC showed a significantly stronger average reduction in MED compared with GER lines, while the reduction in RWC was similar in both subsets in combined stress conditions (Supplementary Table S3). By determining quantum yield of PSII (*F*_v_/*F*_m_) in flag leaves at the first day after stress establishment, we assessed whether extensive damage to the photosynthetic apparatus was detectable 2 d before the same flag leaves were harvested for metabolite analysis. Drought alone did not cause a substantial reduction in *F*_v_/*F*_m_ in either germplasm subset, while in combined stresses, *F*_v_/*F*_m_ decreased to 0.7 for both MED and GER (Fig. 2B), which was significantly lower than in the two other treatments (Supplementary Table S3). This indicates that the combined treatments of drought and heat had a significantly stronger effect on the leaf physiology, and a drop in RWC probably affected the photosynthetic activity of the flag leaf in combined stress conditions. A drop in photosynthetic activity in the leaves under combined drought and heat stress might have secondary effects on metabolite levels that cannot be distinguished from primary effects; therefore, metabolite data from drought-stressed and control leaves will be given priority in the following analyses, while metabolite data from combined stresses will be treated with caution.

In GER lines, continuous drought stress from anthesis to harvest led to a 50% reduction in total biomass, while grain weight was >90% reduced compared with well-watered controls (Fig. 2C, D). Consequently, the treatment explained >60% and 50% of the overall variance in total biomass and grain weight, respectively (Supplementary Table S3). While GER lines showed similar reductions in total biomass under drought and combined drought and heat, MED genotypes showed a significantly stronger reduction in total biomass under combined stresses compared with drought alone (Fig. 2D). Grain weight was only slightly reduced by drought in MED lines (Fig. 2D), leading to a reduction in harvest index of 14% compared with control conditions. In contrast, the harvest index of GER cultivars was decreased by 65% relative to the control. Consequently, grain weight under combined stresses was higher in the MED compared with the GER subpanel. While yield was significantly lower in the MED compared with the GER set under control conditions, it was significantly higher under drought and the combined stresses (Fig. 2C, D). Consequently, the relative reduction in grain weight between control and stress was smaller in the MED than in the GER germplasm set, and absolute grain weight levels were higher under stress in the MED panel.

In summary, the combined stress treatment had a stronger effect on leaf water content, photosynthesis, total biomass, and grain weight compared with drought alone. The two subpanels showed a differential response to stress; the MED lines were characterized by a lower RWC and *F*_v_/*F*_m_, but a higher total biomass and grain number under drought compared with the GER lines. Under the combined stresses, the leaf physiology was not significantly different between the GER and MED lines, but total biomass was significantly lower and grain weight significantly higher in the MED compared with the GER lines.

### Flag leaf metabolite patterns differ between MED and GER in control and drought conditions

**Fig. 3.**
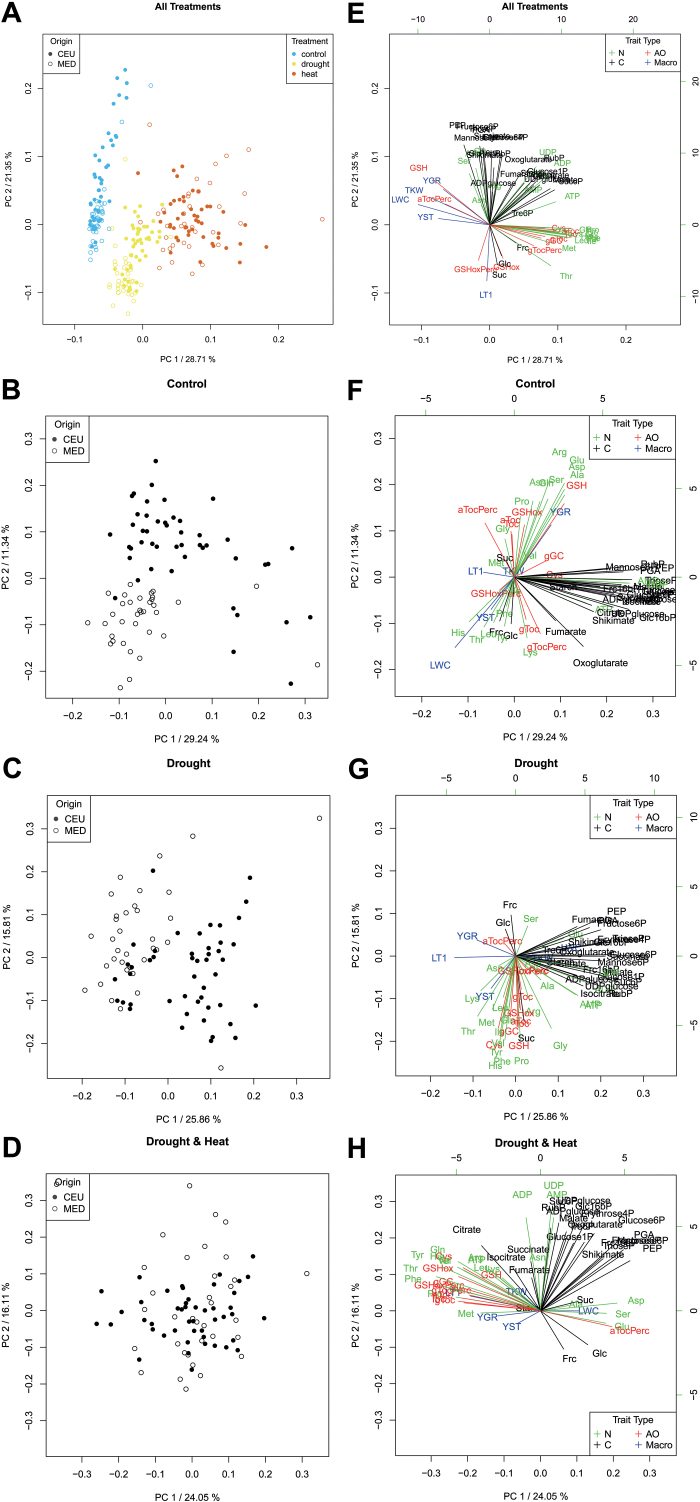
Separation of growth conditions and genotype subpanels by PCA. (A, E) Analysis of the entire data set from 46 GER and 35 MED genotypes including metabolite, morphological, and physiological parameters from all three conditions (control, drought, and combined heat and drought). (B, F) Analysis of metabolite, morphological, and physiological parameters of 46 GER and 35 MED genotypes from control conditions. (C, G) Analysis of metabolite, morphological, and physiological parameters of 46 GER and 35 MED genotypes from drought stress conditions. (D, H) Analysis of metabolite, morphological, and physiological parameters of 46 GER and 35 MED genotypes from combined heat and drought stress conditions. Metabolite data of four replicate flag leaf pools per genotype and treatment sampled 3 d after stress establishment in one of three replicate experiments were used. All other data were taken from three independent experiments, with four replicates per genotype and treatment, each: physiological parameters [PSII intactness (*F*_v_/*F*_m_ value), RWC, and leaf temperature] were quantified between 1 d and 8 d after stress as described in the Materials and methods, while morphological data were determined at final harvest. (A–D) Principal component score plots of principal component 1 (PC1, abscissa) versus PC2 (ordinate) for metabolite data from all three conditions (A), control (B), drought (C), and combined heat and drought (D). Circles indicate the position of the individual genotypes in the plotted two-dimensional space of PC1 versus PC2. GER, filled circles, MED, open circles, in (A) control, blue symbols; drought, yellow symbols; combined heat and drought, red symbols. (E–H) Metabolite loading plots for PC1 and PC2 for data from all three conditions (E), control (F), drought (G), and combined heat and drought (H). Arrow length indicates the loading of metabolite, physiological, and morphological traits onto PC1 (horizontal scale) and PC2 (vertical scale). Nitrogen metabolites (N), green arrows; carbon metabolites (C), black arrows; antioxidants (AO), red arrows; morphological traits (Macro), blue arrows. Amino acids and nucleotides are abbreviated according to the standard three-letter code, Oxoglutarate (α-ketoglutarate), aToc (α-tocopherol), aTocPerc (% α-tocopherol), Erythrose4P (erythrose-4-phosphate), F16bP (fructose-1,6-bisphosphate), Fructose6P (fructose-6-phosphate), Frc (fructose), gGC (γ-glutamyl cysteine), gToc (γ-tocopherol), gTocPerc (% γ-tocopherol), G16bP (glucose-1,6-bisphosphate), Glucose1P (glucose-1-phosphate), Glucose6P (glucose-6-phosphate), Glc (glucose), GSH (glutathione), GSHoxPerc (% glutathione oxidized), Mannose6P (mannose-6-phosphate), PEP (phospho*enol* pyruvate), PGA (3-phosphoglycerate), RubP (ribulose-1,5-bisphosphate), Shik (shikimate), Sucrose6P (sucrose-6-phosphate), Suc (sucrose), Toc (total Tocopherol), TriP (triose-phosphates), Tre6P (trehalose-6-phosphate), UDPglc (UDP-glucose). LWC (relative leaf water content), LT1 (leaf temperature), YBM (total biomass), YST (straw biomass), YGR (grain yield).

**Fig. 4.**
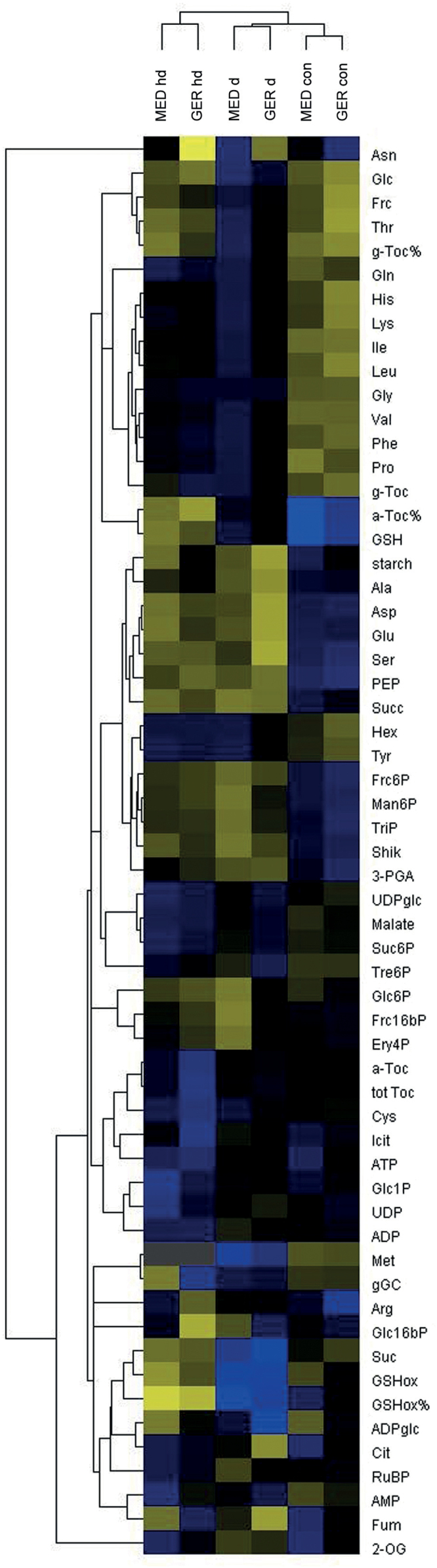
Hierarchical cluster analysis (HCA) of metabolite data. Mean values for metabolite data from 3 d after stress establishment in all three conditions were calculated separately for all 46 GER and all 35 MED genotypes prior to HCA. The false-color scale designates blue fields as below the median and yellow fields as above the median. con, control; d, drought; hd, combined heat and drought. Amino acids and nucleotides are abbreviated according to the standard three-letter code, 2-OG (α-ketoglutarate), a-Toc% (α-tocopherol), a-Toc% (% α-tocopherol), ADPglc (ADP-glucose) Cit (citrate), Ery4P (erythrose-4-phosphate), F16bP (fructose-1,6-bisphosphate), F6P (fructose-6-phosphate), Frc (fructose), Fum (fumarate), gGC (γ-glutamyl cysteine), g-Toc (γ-tocopherol), g-Toc% (% γ-tocopherol), G16bP (glucose-1,6-bisphosphate), G1P (glucose-1-phosphate), G6P (glucose-6-phosphate), Glc (glucose), GSH (glutathione), %GSH red (% glutathione reduced), Icit (isocitrate), Man6P (mannose-6-phosphate), PEP (phospho*enol* pyruvate), 3-PGA (3-phosphoglycerate), Pyr (pyruvate), RubP (ribulose-1,5-bisphosphate), Shik (shikimate), Suc6P (sucrose-6-phosphate), Suc (sucrose), Succ (succinate), tot Toc (total tocopherol), TriP (triose-phosphates), Tre6P (trehalose-6-phosphate), UDPglc (UDP-glucose).

Metabolite profiles of control, drought-stressed, as well as heat- and drought-stressed flag leaves were clearly separated by PCA and hierarchical cluster analysis (HCA) (Figs 3A, 4). While most free amino acids show strong positive loading on principal component1 (PC1), most physiological and morphological traits, together with the antioxidants α-tocopherol and glutathione, exhibit a negative loading on PC1 (Fig. 3E). Interestingly, separation of the three conditions still occurred when morphological data were omitted from the PCA (Supplementary Fig. S3), indicating that metabolite data determined the separation by PCA. When the clustering of metabolites was analyzed for each treatment separately, metabolite profiles of GER and MED accessions fell into different but overlapping clusters in control conditions and drought stress (Fig. 3B, C), but not in combined stress (Fig. 3D). The separation of GER and MED was predominantly conferred by free major amino acids and antioxidants in control conditions (Fig. 3F). Under drought, however, the two subpanels were discriminated by minor amino acids and sucrose (Fig. 3G). The loading of the individual metabolites on PC1 and PC2 is different in control and drought (Fig. 3F, G). This indicates that (i) metabolite patterns differed between both treatments and (ii) metabolic adaptation of GER and MED to drought stress relies on different metabolic strategies.

We therefore examined more closely (i) how metabolic adaptation to drought and combined stresses can be distinguished; (ii) which metabolic changes under drought and combined drought and heat were common to GER and MED; and (iii) in what respect the metabolite profiles in GER and MED differed in control, drought, and combined stress conditions.

### Combined stress leads to a strong accumulation of free minor amino acids, while major amino acid pools are diminished by drought in the GER and MED subpanels

**Fig. 5.**
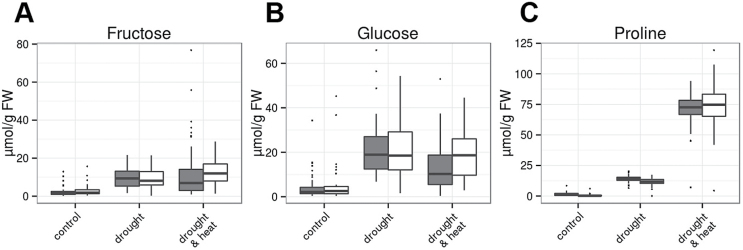
Steady-state contents of hexoses and proline in flag leaves at 3 d after stress establishment. (A) Fructose; (B) glucose; (C) proline. Data of four replicates per genotype and treatment from 46 GER lines (gray bars) and 35 MED lines (white bars) were grouped for analysis and are shown from control (left), drought stress (middle), and combined heat and drought (right). In the box plots, the median± upper and lower quartile are indicated as boxes, while whiskers indicate values within 1.5 times the interquartile range away from the median from the lower and upper quartile. Dots reflect outliers of these regions (Tukey, 1977). Results of the ANOVA are provided in Supplementary Table S3. (This figure is available in colour at *JXB* online.)

First, we investigated changes in flag leaf metabolite content under drought and combined stresses compared with control conditions that were similar in MED and GER subpanels. HCA revealed that the accumulation of a considerable number of metabolites was highest in combined stresses and less pronounced in drought stress compared with control conditions in both GER and MED lines (Fig. 4; Supplementary Figs S4B, S5). Besides the compatible solute proline, the two major amino acids glutamine and glycine as well as tocopherols and seven minor amino acids belonged to this group of metabolites (Supplementary Fig. S5). Free hexoses that commonly serve as vacuolar osmolytes were increased to similar levels under drought and combined drought and heat stress compared with control conditions (Fig. 5C). The major amino acids aspartate, glutamate, and serine, as well as starch showed a specific reduction under drought, but not in combined stresses as compared with control conditions (Supplementary Figs S4B, S6). Taken together, a large number of metabolites showed responses specific to drought and combined stresses independently of the germplasm group. This suggests that both stresses elicit qualitatively and quantitatively different responses in barley.

### Major carbon metabolism responds differently to drought in GER and MED genotypes

**Fig. 6.**
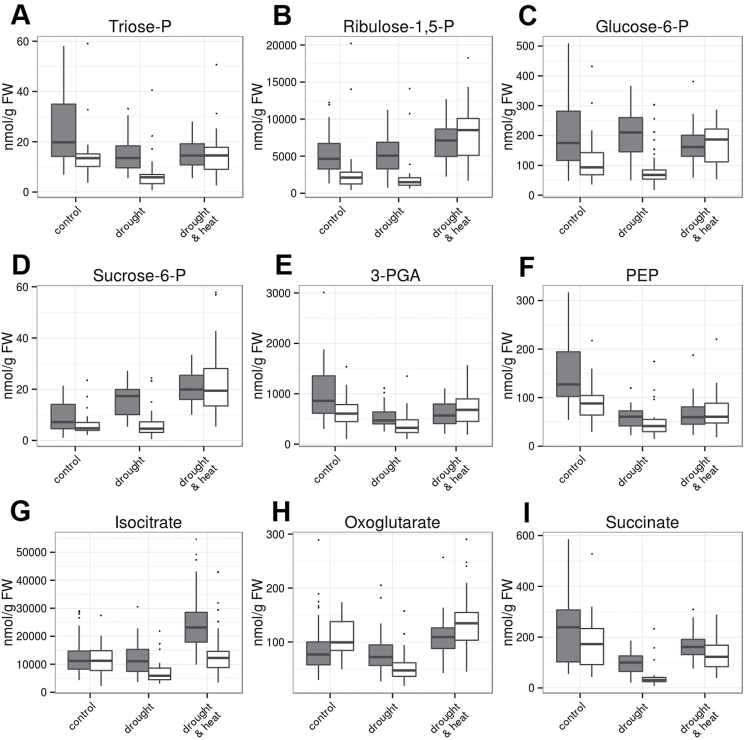
Steady-state contents of phosphorylated and carboxylate intermediates in flag leaves at 3 d after stress establishment. (A) Triose-phosphates; (B) ribulose-1,5-bisphosphate; (C) glucose-6-phosphate; (D) sucrose-6-phosphate; (E) isocitrate; (F) 2-oxoglutarate; (G) succinate; (H) 3-phosphoglycerate; (I) phosphoenolpyruvate. Data of four replicates per genotype and treatment from 46 GER lines (gray bars) and 35 MED lines (white bars) were grouped for analysis and are shown from control (left), drought stress (middle), and combined heat and drought (right). In the box plots, the median± upper and lower quartile are indicated as boxes, while whiskers indicate values within 1.5 times the interquartile range away from median from the lower and upper quartile. Dots reflect outliers of these regions (Tukey, 1977). Results of the ANOVA are provided in Supplementary Table S3. (This figure is available in colour at *JXB* online.)

**Fig. 7.**
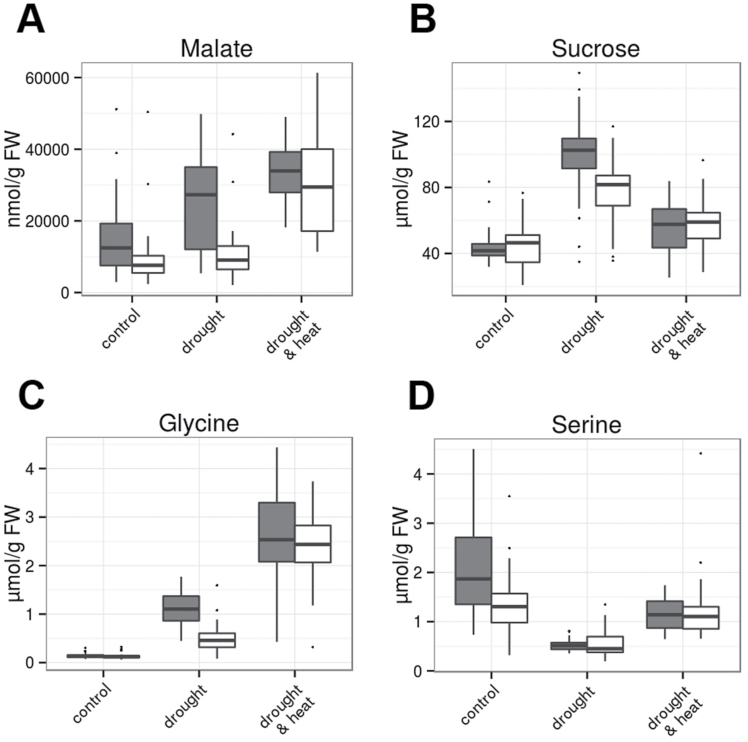
Steady-state contents of major metabolites in flag leaves at 3 d after stress establishment. (A) Malate; (B) sucrose; (C) glycine; (D) serine. Data of four replicates per genotype and treatment from 46 GER lines (gray bars) and 35 MED lines (white bars) were grouped for analysis and are shown from control (left), drought stress (middle), and combined heat and drought (right). In the box plots, the median± upper and lower quartile are indicated as boxes, while whiskers indicate values within 1.5 times the interquartile range away from median from the lower and upper quartile. Dots reflect outliers of these regions (Tukey, 1977). Results of the ANOVA are provided in Supplementary Table S3. (This figure is available in colour at *JXB* online.)

The metabolite pattern in combined drought and heat stress was largely indistinguishable between GER and MED by PCA (Fig. 3D), which can be explained by the fact that steady-state levels of most metabolites were comparable between the GER and MED subpanels (Figs 5–7; Supplementary Figs S5, S6). In contrast, the response of major carbon metabolism to drought differed between these two groups of genotypes at 3 d after stress establishment. Foliar levels of phosphorylated intermediates involved in the Calvin cycle and sucrose biosynthesis, such as triose-phosphates, ribulose-1,5-bisphosphate, glucose-6-phosphate, and sucrose-6-phosphate, were elevated in GER compared with MED already in control conditions (Fig. 6A–D). Upon drought stress, the contents of these intermediates remained largely unaltered in GER cultivars, while they decreased further in MED lines (Fig. 6A). Consequently, genotype subset explained >10% of the overall variance for these intermediates (Supplementary Table S3). In parallel, foliar pools of the tricarboxylic acid (TCA) cycle intermediates isocitrate, oxoglutarate, and succinate were much more depleted in MED compared with the GER subpanel in drought stress conditions (Fig. 6E–G; Supplementary Table S3). In summary, MED accessions exhibited depleted pools of phosphorylated intermediates in major carbon and anaplerotic carboxylate metabolism in response to drought at 3 d after stress establishment, while this metabolic adaptation was less pronounced in GER cultivars. This indicates that MED genotypes, but not GER cultivars, have reduced metabolic activity at 3 d after stress establishment. In support of this assumption, an increased glycine/serine ratio in drought-stressed GER flag leaves compared with drought-stressed MED leaves and GER control leaves (Fig. 7C, D) indicates elevated photorespiratory flux in drought-stressed GER leaves. Elevated photorespiration is caused by high Calvin cycle activity during unfavorable conditions such as drought stress, when CO_2_ availability is low.

Sucrose and malate, both representing transitory carbon storage compounds in the vacuole, accumulated more strongly in drought-stressed GER than in drought-stressed MED genotypes (Fig. 7A, B), while pool sizes of phosphorylated C_3_-compounds such as 3-phosphoglycerate (3-PGA) and phospho*enol* pyruvate (PEP) decreased much more strongly in GER than in MED upon drought stress (Fig. 6H, I).

Since the geographic origin of the investigated MED genotypes is quite heterogeneous, we assessed whether the observed response of major carbon and carboxylate metabolism to drought stress (see Fig. 6) can be distinguished between MED lines of different geographic origin. This was not the case, as the accumulation of most of these intermediates was comparable between all MED subpanels under drought stress (Supplementary Figs S7, S8). Consistent with the data depicted in Fig. 6, flag leaf contents of triose-phosphates, ribulose-1,5-bisphosphate, glucose-6-phosphate, and sucrose-6-phosphate were elevated in GER in comparison with all MED subpanels in both drought stress and control, while malate, succinate, and glycine contents were increased only in drought-stressed GER flag leaves relative to the individual MED subpanels (Supplementary Figs S7, S8). In contrast to intermediates in major carbon metabolism, flag leaf contents of major N metabolites such as glutamine and asparagine showed substantial differences between MED lines of different geographic origin in all three growth conditions (Supplementary Fig. S9), which may reflect the genetic heterogeneity of organic N metabolism in the MED subpanel.

### Association genetics reveal mQTLs for flag leaf glutathione and succinate content that co-localize with genes involved in their biosynthesis

As we detected significant genetic effects on metabolite levels under different treatments, our objective was to identify genomic regions affecting metabolite levels. In order to identify mQTLs, we selected 2596 of the 3967 mapped SNP markers represented on the Illumina 9K SNP array (Close *et al.*, 2009) that had a minor allele frequency of at least 10% and maximum missing data frequency of 10% in the entire population. By using the selected SNP data and the entire metabolite data set of all 81 examined genotypes from all three growth conditions, we calculated genome-wide associations in a mixed linear model in which population structure was corrected by a Kinship matrix.

Our genome-wide association study (GWAS) revealed significant mQTLs for 25 metabolites, involving a total of 566 SNP markers. The number of SNP markers significantly associated with metabolite levels ranged from 1 to 116 per metabolite. Of all significant mQTLs, 13 were supported by >10 adjacent SNP markers with a *P*-value <0.01 each (Table 1).

**Table 1.** Overview over the detected mQTLs in the barley genome mQTLs unique for one metabolite are indicated in italics, while mQTLs that co-localize with structural genes of the corresponding pathways are shown in bold. The SNPs that were most significantly associated with individual metabolite contents at the respective mapping position are included.

**Chromosome**	**Position (cM**)	**Metabolite**	**Most significant SNP**	**Gene model**	**Annotation**
**2H**	50.9–67.9 53.7–62.7	Glutathione Starch	i_SCRI_RS_146010 i_SCRI_RS_144776		
**3H**	107.8–108.4 107.8–108.6	Glutathione Starch	i_SCRI_RS_138723 i_SCRI_RS_138723		
**5H**	44–44.5 **44.2** 44.2 *47.7–48.1*	Starch **Glutathione** Succinate *Glycine*	i_SCRI_RS_103377 i_SCRI_RS_103377 i_SCRI_RS_169826 i_SCRI_RS_144696	AK354495	Glutathione synthase (GS)
**6H**	47.9–53.5 **52.5–53.5**	Glutathione **Succinate**	i_SCRI_RS_195226 i_SCRI_RS_158873	MLOC_62667	Succinate semialdehyde DH (SSADH)
**7H**	*61.5–70.3* *120.8–125.3* *118.3–125.3*	*Glutathione* *%α-Tocopherol* *γ-Tocopherol*	i_SCRI_RS_200107 i_SCRI_RS_4520 i_SCRI_RS_4520	MLOC_34476 MLOC_34476	Homogentisate phytyl transferase (HPT) Homogentisate phytyl transferase (HPT)

**Fig. 8.**
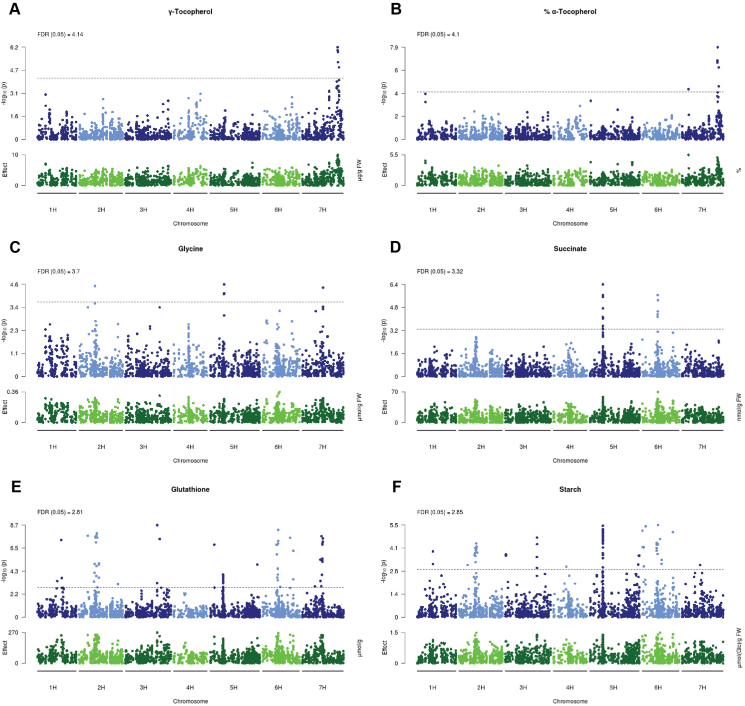
Manhattan plots showing the genome-wide association of flag leaf metabolite contents with SNP markers. Manhattan plots for the association analysis of the metabolic traits γ-tocopherol (A), relative flag leaf α-tocopherol (B), glycine (C), succinate (D), glutathione (E), and starch (F) from a GWAS over all treatments (as fixed effect). Each dot along the *x*-axis represents the negative logarithmic *P*-value (blue) and effect size (green) of a single SNP marker.

Data are taken from Fig. 8

Single mQTLs for γ-tocopherol, tocopherol pool composition (% α-tocopherol), and glycine were identified (Fig. 8A–C), while more than one mQTL was detected for succinate, glutathione, and starch (Fig. 8D–F). Interestingly, most of the identified mQTLs co-localize with other detected mQTLs in the genome (Table 1). For instance, the region between 50.9 cM and 62.7 cM on chromosome 2H is associated with flag leaf glutathione and starch, while the region around 44.2 cM on chromosome 5H is associated with flag leaf starch, glutathione, and succinate contents (Table 1).

At first glance, it seems very unlikely that structural genes of the corresponding biosynthetic pathways co-localize in these two regions. However, one of the two annotated glutathione synthases (AK354495 on morex_contig_45036) maps exactly to 44.2 cM on chromosome 5H (http://pgsb.helmholtz-muenchen.de/plant/barley/ga/searchjsp/index.jsp;International Barley Genome Sequencing Consortium, 2012), indicating a functional connection between this glutathione synthase (GS) isoform and flag leaf glutathione contents. The other GS isoform (AK364878) maps to 98.3 cM on chromosome 7H, which is almost 30 cM apart from the specific mQTL for flag leaf glutathione content on chromosome 7H (Table 1), indicating that this second GS isoform is not related to the mQTL for flag leaf glutathione content on chromosome 7H.

The chromosomal region between 47.9 cM and 53.5 cM on chromosome 6H harbors two mQTLs, one for flag leaf glutathione and one for flag leaf succinate content (Table 1). Interestingly, the only isoform of succinate semialdehyde dehydrogenase (SSADH) that is currently annotated in the barley genome (MLOC_62667) is located at 52.9 cM on chromosome 6H (http://pgsb.helmholtz-muenchen.de/plant/barley/ga/searchjsp/index.jsp;International Barley Genome Sequencing Consortium, 2012), which is within the range of the mQTL. SSADH is required for the operation of the γ-aminobutyric acid (GABA) shunt (Bouche *et al.*, 2003; Ludewig *et al.*, 2008) that scavenges reactive oxygen species (ROS) in abiotic stress situations, such as heat, drought, and UV-B (Bouche *et al.*, 2003; Ludewig *et al.*, 2008; Liu *et al.*, 2011).

Our GWAS also revealed a specific mQTL for flag leaf glycine content at around 48 cM on chromosome 5H (Table 1). Based on the current genome annotation, we were unable to identify genes that code for enzymes of the photorespiratory C_2_-cycle at this chromosomal position. Since glycine accumulation is predominantly controlled by photorespiratory flux, the identified mQTL at 48 cM on chromosome 5H might influence glycine contents indirectly.

### A detected mQTL for flag leaf γ-tocopherol content on chromosome 7H co-localizes with the major barley homogentisate phytyl transferase isoform

**Fig. 9.**
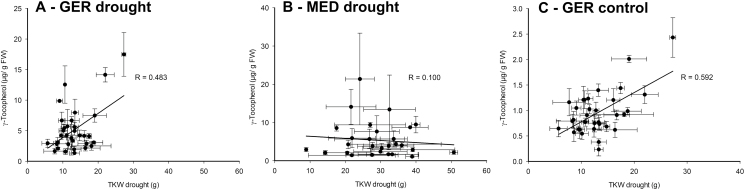
Correlation between TKW in terminal drought stress and flag leaf γ-tocopherol content at 3 d after stress establishment. TKW in terminal drought stress of 42 GER elite breeding lines (A and C) and 35 MED accessions (B) is plotted against flag leaf γ-tocopherol content of the same genotypes at 3 d after stress establishment in various conditions. Correlation between TKW under drought stress and γ-tocopherol content of drought-stressed flag leaves at 3 d after stress establishment in 42 GER elite lines (A), correlation between TKW under drought stress and γ-tocopherol content of drought-stressed flag leaves at 3 d after stress establishment in 35 MED genotypes (B), and correlation between TKW under drought stress and flag leaf γ-tocopherol content under control conditions in 42 GER elite lines (C). The correlation coefficient *R* was calculated based on 42 data points in (A) and (C) and for 35 data points in (B), and is given in the diagrams. Each data point represents the mean ±SE of four replicates.

As described above, our GWAS revealed an mQTL for flag leaf γ-tocopherol content and tocopherol pool composition (i.e. % α-tocopherol) between 118.3 cM and 125.3 cM on chromosome 7H (Fig. 8A, B). Interestingly, MLOC_34476.3 (located on morex_contig_2548480), coding for one of the two barley homogentisate phytyl transferases (HPTs) that catalyze the committed step of tocopherol biosynthesis, maps at 121.8 cM on chromosome 7H of the current barley genome assembly available at http://pgsb.helmholtz-muenchen.de/plant/barley/ga/searchjsp/index.jsp. Therefore, our data strongly suggest that the HPT isoform on the long arm of chromosome 7H is causally connected to the detected mQTL for flag leaf γ-tocopherol content. Interestingly, a correlation of flag leaf γ-tocopherol content at 3 d after stress establishment in drought stress with TKW in drought stress was evident within the 36 elite breeding lines in the GER panel (Fig. 9A), but absent from the MED collection (Fig. 9B). Furthermore, γ-tocopherol content of elite line flag leaves grown under control conditions correlated with the TKW of these lines under drought stress with *R*>0.59 (Fig. 9C), indicating that foliar γ-tocopherol content in control conditions, albeit much lower than in drought-challenged plants, might be predictive for TKW of elite breeding lines under stress. This indicates a potential link between foliar γ-tocopherol content and grain filling under drought stress in GER elite lines, which requires further investigation.

In summary, our study has revealed three mQTLs that co-localize with candidate genes involved in antioxidant defense (tocopherol and glutathione biosynthesis) and ROS scavenging (GABA shunt). As we have exclusively identified mQTLs for metabolites that are involved in stress protection, this demonstrates the power of our GWAS approach that used metabolite data from two abiotic stress conditions.

## Discussion

### Proline accumulation does not differ between GER and MED genotypes while sucrose and malate seem to represent major osmolytes in breeding lines after 5 d of drought stress

Abiotic stresses such as cold, freezing, salinity, heat, and drought eventually result in reduced water availability and, therefore, tolerance mechanisms towards particular abiotic stress situations involve distinct as well as common responses. To date, it is widely accepted that the accumulation of osmolytes and compatible solutes improves abiotic stress tolerance in plants (for a recent review, see Krasensky and Jonak, 2012). Not all plant species accumulate the same range of osmolytes and compatible solutes. For instance, trehalose as an osmolyte and glycine betaine as a compatible solute are only produced by a limited number of plant species (Krasensky and Jonak, 2012, and references cited therein). Furthermore, the effectiveness of individual osmolytes and compatible solutes depends on the type of abiotic stress that the plants experience.

The amino acid proline serves as compatible solute and is derived from glutamate in a three-step reaction. Proline accumulation is commonly observed in drought, salt, and heat stress in a wide range of plants, but proline accumulation of individual plant species in response to abiotic stress can vary. For instance, Arabidopsis and tobacco plants do not accumulate proline in heat stress or combined heat and drought stress (Rizhsky *et al.*, 2004; Dobra *et al.*, 2010; Prasch and Sonnewald, 2013), and it has been reported that proline may be toxic in heat stress conditions (Lv *et al.*, 2011). For barley, a connection between foliar proline accumulation and yield stability in field trials in heat- and drought-prone environments has been observed (Singh *et al.*, 1972). On average, we observed a >10-fold accumulation of proline at 3 d after stress establishment in drought and a >60-fold accumulation of proline in combined heat and drought stress. However, the response of drought-adapted MED genotypes and GER cultivars bred for optimal yield in temperate climate was comparable, indicating that proline accumulation represents a crucial and conserved response in the investigated barley genotypes. Therefore, the adaptation to drought or combined stresses in the investigated collection cannot differ based on proline contents alone.

Consistent with previous studies in other species (for a compilation, see Krasensky and Jonak, 2012), we have observed diminished starch contents in drought-stressed barley flag leaves compared with controls. Functional studies in Arabidopsis have demonstrated that starch mobilization provides sugar moieties for osmolyte accumulation in mesophyll cells (Kaplan and Guy, 2004; Thalmann *et al.*, 2016) as well as for stomatal opening in guard cells (Prasch *et al.*, 2015; Horrer *et al.*, 2016). It has been shown that the mobilization of starch by amylases plays a vital role for osmotic stress adaptation in Arabidopsis (Kaplan and Guy, 2005; Thalmann *et al.*, 2016), while loss of the major amylase of guard cells improves drought tolerance due to impaired stomatal opening (Prasch *et al.*, 2015; Horrer *et al.*, 2016). Flag leaf starch contents as well as hexose accumulation did not differ significantly between GER and MED genotypes after 5 d of drought stress. However, sucrose and malate showed a significantly stronger accumulation in drought-stressed flag leaves of GER cultivars compared with MED accessions. This observation might explain an elevated RWC in GER flag leaves in comparison with the MED flag leaves after 11 d of drought stress, since it has been described that, besides soluble sugars, barley vacuoles can contain high concentrations of malate (Tohge *et al.*, 2011). Increased vacuolar malate concentrations in GER cultivars may, in turn, reduce water loss during drought stress.

Taken together, our study provides an indication that sucrose and malate may serve as additional major osmolytes in barley during drought stress adaptation in addition to hexoses.

### MED genotypes show a more pronounced adaptation of flag leaf metabolic activity upon drought stress

In contrast to osmolyte and compatible solute accumulation, not much is known about the influence of intermediates in major carbon and nitrogen metabolism on abiotic stress tolerance. Commonly, an increase in free amino acid content is observed in abiotic stress (summarized by Krasensky and Jonak, 2012, and references therein). It is not clear, however, if this is based on increased *de novo* biosynthesis of amino acids or due to stress-dependent protein breakdown. Consistent with the literature, we have observed an accumulation of most minor amino acids in drought and combined heat and drought stress, especially branched-chain and aromatic amino acids. Since minor amino acids are predominantly affected, this favors the idea that protein breakdown is responsible for the observed increase in free amino acid levels.

A few reports exist to date on the response of carboxylate pool on abiotic stress. Conflicting data exist for drought-stressed Arabidopsis plants. While it was observed that the pool sizes of some carboxylates increased upon artificial dehydration (Urano *et al.*, 2009), malate and citrate contents decreased in another study investigating drought and combined heat and drought stress in controlled growth regimes (Prasch and Sonnewald, 2013). Consistently, tomato plants constitutively overexpressing the abscisic acid (ABA)-responsive transcription factor *Sl*AREB1 were more tolerant to salinity and drought (Orellana *et al.*, 2010) and exhibited decreased malate and citrate contents in leaves (Bastías *et al.*, 2014). In our study, flag leaves of drought-adapted MED accessions contained significantly less isocitrate, oxoglutarate, succinate, and malate contents after 5 d of drought stress compared with GER genotypes. It is tempting to speculate that diminished carboxylate pool sizes might be associated with reduced metabolic activity as an adaptation to drought. Isocitrate and oxoglutarate serve an anaplerotic function as carbon backbones for nitrogen assimilation (Scheible *et al.*, 2000, Dutilleul *et al.*, 2005; Lemaitre *et al.*, 2007), indicating that nitrogen assimilation might be dampened in drought-stressed MED flag leaves in comparison with drought-stressed GER flag leaves. In addition, the pools of phosphorylated intermediates in major carbon metabolism were depleted in MED lines in response to drought, which was less pronounced or absent in GER cultivars. Taken together, this indicates that MED genotypes may have already reduced the capacity of major carbon and nitrogen metabolism at 3 d after stress establishment, which does not occur to the same extent in GER genotypes. Larger pools of metabolic intermediates are likely to be associated with the stay-green phenotype of most elite breeding lines that tend to retain photosynthetic competence during intermittent stress (Thomas and Horwarth, 2000). This idea is also supported by the observation that drought-stressed GER flag leaves exhibit an increased glycine/serine ratio compared with GER control leaves and drought-stressed MED leaves, which is a clear indicator of elevated photorespiratory flux (Novitskaya *et al.*, 2002; Kebeish *et al.*, 2007). High rates of photorespiration are caused when the Calvin cycle operates during unfavorable conditions such as drought stress, when CO_2_ availability is low, which might be avoided by a drought-responsive attenuation of major carbon and nitrogen metabolism in MED flag leaves. In addition, organic nitrogen may be relocated from stressed leaves to developing spikes, as demonstrated for barley and maize leaves during natural senescence (Martin *et al.*, 2006; Habash *et al.*, 2007).

### The detected mQTLs for flag leaf glutathione and γ-tocopherol content might play a role for morphological traits

Our study has revealed three mQTLs that co-localize with candidate genes involved in antioxidant defense and ROS scavenging. Since we have performed a GWAS with metabolite data obtained from two abiotic stress conditions, the fact that we have exclusively identified mQTLs for metabolites that play a role in stress protection demonstrates the power of our approach.

Interestingly, one of the detected mQTLs for flag leaf glutathione content, that is located between 61.5 cM and 70.3 cM on chromosome 7H, co-localizes with a QTL for the number of kernels per ear at 70.7 cM on chromosome 7H (Supplementary Fig. S10). In our data set, this is the only co-localization of mQTLs with QTLs for morphological and physiological traits. Since genes involved in glutathione metabolism have not yet been annotated to this region, this observation requires further investigation. So far, a co-localization of mQTLs with QTLs for morphological traits has only been achieved in two studies using 287 and 197 recombinant inbred maize lines, respectively (Riedelsheimer *et al.*, 2012; Wen *et al.*, 2015).

In addition, a weak correlation between flag leaf γ-tocopherol content and TKW in drought stress was evident in elite breeding lines. In transgenic tobacco, it has been demonstrated that increased γ-tocopherol content improves tolerance towards osmotic and oxidative stress (Abbasi *et al.*, 2007), substantiating a potential functional connection between flag leaf γ-tocopherol content and TKW in drought stress. However, this correlation could not be observed when data from all genotypes or only from the MED subpanel were plotted. While the variablity of flag leaf γ-tocopherol content was similar in drought-stressed GER and MED subpanels, more than half of the MED accessions showed higher TKW than the best elite line under drought stress (compare Fig. 9A and B). This may explain why the correlation between flag leaf γ-tocopherol content and TKW was absent when the entire data set was analyzed. On the other hand, only three genotypes exhibited >10 µg of γ-tocopherol g^–1^ FW in each subpanel, indicating that the observed correlation is based on only very few genotypes. It will be necessary to investigate additional genotypes to verify this observation.

The identified mQTL for flag leaf γ-tocopherol content between 118.3 cM and 125.3 cM was computed with data from all 81 accessions and is statistically significant. One of the two barley HPT genes (MLOC_37476), catalyzing the committed step of tocopherol biosynthesis, is located at 121.8 cM of the current barley genome assembly (http://pgsb.helmholtz-muenchen.de/plant/barley/ga/searchjsp/index.jsp;International Barley Genome Sequencing Consortium, 2012). Since the other barley HPT gene located at 132.5 cM on chromosome 2H is currently not supported by ESTs and RNAseq tags, we assume that the identified gene codes for the major HPT isoform in leaves. Using tocochromanol data from 1534 field-grown spring barley accessions from the Barley Coordinated Agricultural Project (CAP), Graebner *et al.* (2015) have recently identified several mQTLs for grain γ-tocopherol, α-tocotrienol, β-tocotrienol, γ-tocotrienol, δ-tocopherol, and δ-tocotrienol content between 136 cM and 145 cM on the long arm of chromosome 7H. However, Graebner *et al*. used the USDA linkage map generated by Muñoz-Amatriaín *et al.* (2014), that localized the HPT gene of interest (MLOC_37476) at 138 cM of chromosome 7H. In conclusion, our study has identified the same HPT gene, MLOC_37476, to be associated with flag leaf γ-tocopherol content that was earlier shown to be associated with barley grain tocochromanol content by Graebner *et al.* (2015).

Since HPT catalyzes the committed step in tocopherol biosynthesis, it seems difficult to explain why our study has not identified an association of the same mQTL with flag leaf α-tocopherol content, which represents the major tocochromanol species in barley leaves. However, overexpression of HPT in Arabidopsis was shown to increase the total tocopherol pool size by up to 4.4-fold, while the γ-tocopherol pool was elevated up to 15-fold (Collakova and DellaPenna, 2003*a*), indicating (i) a stronger effect of increased HPT activity on γ-tocopherol content and that (ii) γ-tocopherol methyl transferase (γ-TMT), the ultimate step in α-tocopherol synthesis, is limiting for α-tocopherol accumulation. When Arabidopsis HPT overexpressors were subjected to abiotic stress, the strongest accumulation was observed for γ-tocopherol (Collakova and DellaPenna, 2003*b*), indicating that γ-TMT activity is also limiting for α-tocopherol accumulation under stress. Similarly, tocopherol pool composition was altered in favor of γ-tocopherol in barley leaves subjected to drought or combined stresses, indicating that barley γ-TMT activity may also be limiting for α-tocopherol accumulation in abiotic stress.

### An mQTL for flag leaf succinate content seems to be linked to a gene coding for the GABA shunt enzyme SSDAH

Besides being an intermediate in the anaplerotic TCA cycle, succinate is involved in the so-called GABA shunt, which is required to ameliorate ROS load under abiotic stress conditions in Arabidopsis and tobacco (Bouche *et al.*, 2003; Ludewig *et al.*, 2008; Liu *et al.*, 2011). In the GABA shunt, GABA is converted to succinate by the consecutive action of GABA transaminase (GABA-T) and SSADH, producing NADH in the mitochondrial matrix, where GABA-T and SSADH are localized (see Ludewig *et al.*, 2008, and references therein). Thereby, the GABA shunt bypasses the TCA cycle enzymes oxoglutarate dehydrogenase and succinyl-CoA ligase.

Our study has identified an mQTL for flag leaf succinate content between 52.5 cM and 53.5 cM on chromosome 6H, and the only SSADH gene currently annotated in the barley genome (MLOC_62667) also maps to 52.9 cM on chromosome 6H, indicating a potential functional connection between SSADH and flag leaf succinate contents. In ABA-deficient Arabidopsis mutants, a transcriptional induction of GABA-T isoform GAD4 coincided with a stronger increase in GABA and succinate contents upon dehydration compared with the wild type (Urano *et al.*, 2009). In contrast, we have observed that succinate contents decreased in drought-stressed barley flag leaves compared with well-watered controls, while GABA contents did not change upon drought stress. Similarly, GABA and succinate did not accumulate in leaves of tomato plants constitutively overexpressing the ABA-responsive transcription factor *Sl*AREB1 (Bastías *et al.*, 2014). Unfortunately, no information on the expression of GABA shunt genes is available for drought-stressed barley and the tomato transgenics studied by Bastías *et al.* (2014). Therefore, we cannot decide if the transcriptional activation of the GABA shunt or the metabolic flux through the shunt differs between the investigated plant species.

## Conclusion

Our study revealed differences in metabolic adaptation strategies to drought stress between German elite breeding lines and barley accessions adapted to drought-prone Mediterranean climate. Drought-adapted barley genotypes attenuated major leaf carbon metabolism much more strongly than elite lines during drought stress adaptation, which may cause increased rates of photorespiration in leaves of drought-stressed elite lines. In parallel, elite breeding lines exhibited a stronger accumulation of sucrose and malate, which may serve as osmolytes, and retained a higher RWC than exotic barley accessions when subjected to drought stress.

Using a GWAS with flag leaf metabolite data from control and two different abiotic stress conditions in barley, we have identified three mQTLs for metabolites involved in antioxidative defence that co-localize with genes of the corresponding pathways. For flag leaf γ-tocopherol content, a weak correlation with TKW under drought stress was observed in a particular subset of the analyzed barley genotypes that is used for breeding. In addition, one detected mQTL for flag leaf glutathione content co-localized with a QTL for kernel number per ear. In the future, it will be challenging to identify the genetic basis of the identified mQTLs and to investigate the contribution of the identified candidate genes to antioxidant capacity and agronomic performance in abiotic stress conditions.

## Supplementary Material

Supplementary Data
