# Supplementary material for: Metabolite profiling of barley flag leaves under drought and combined heat and drought stress reveals metabolic QTLs for metabolites associated with antioxidant defense
Source: J Exp Bot. 2017 Mar 10;68(7):1697–713. doi: 10.1093/jxb/erx038 (PMC5441916; doi:10.1093/jxb/erx038)
Supplement: Supplementary Data [file erx038_Supplementary_Data.zip › supplementary_Table_S1_Figures_S1_S10.pdf]

SUPPLEMENTARY DATA

Figure S1. Linkage disequilibrium in the investigated barley germplasm collection.

Figure S2. Principal component analysis of the genetic structure of the investigated barley collection.

Figure S3. PCA based on metabolite data from all three growth regimes.

Figure S4. Hierarchcical cluster analysis (HCA) of metabolite data with highlighted subclades.

Figure S5. Metabolites that accumulate stronger in flag leaves under combined than in drought stress.

Figure S6. Metabolites with diminished steady state level in drought stressed flag leaves.

Figure S7. Steady state contents of phosphorylated intermediates in flag leaves at 3 days after stress establishment by geographic origin.

Figure S8. Steady state contents of carboxylates and amino acids in flag leaves at 3 days after stress establishment by geographic origin.

Table S1. Barley genotypes investigated in this study.

Table S2. Compilation of flag leaf metabolite data from control, drought and combined stress. (TableS2metabolitedata.xlsx)

Table S3. ANOVAs on metabolite, physiological and morphological data. (TableS3anovas.xlsx)

Supplementary Tables

Table S1. Barley genotypes investigated in this study.

[*] Proprietary breeding lines under exclusive license.

[§] To synchronize flowering between all genotypes, these lines have been cultivated in short days for 4 weeks prior to the transfer to long day conditions.

Genotype	Geographic origin	Subset Classification	K-means cluster	Cultivar Type	Inflorescence type
Auriga	Germany	GER	1	Cultivar	2-rowed
B3094	Germany	GER	1	Breeding*	2-rowed
B3117	Germany	GER	1	Breeding*	2-rowed
B3162	Germany	GER	1	Breeding*	2-rowed
B3163	Germany	GER	1	Breeding*	2-rowed
B3194	Germany	GER	1	Breeding*	2-rowed
B3250	Germany	GER	1	Breeding*	2-rowed
B3254	Germany	GER	1	Breeding*	2-rowed
B3274	Germany	GER	1	Breeding*	2-rowed
B3298	Germany	GER	1	Breeding*	2-rowed
B3332	Germany	GER	1	Breeding*	2-rowed
B3457	Germany	GER	1	Breeding*	2-rowed
B3460	Germany	GER	1	Breeding*	2-rowed
B3482	Germany	GER	1	Breeding*	2-rowed
B3484	Germany	GER	1	Breeding*	2-rowed
B3542	Germany	GER	1	Breeding*	2-rowed
B3684	Germany	GER	1	Breeding*	2-rowed
B3813	Germany	GER	1	Breeding*	2-rowed
B3828	Germany	GER	1	Breeding*	2-rowed
B3862	Germany	GER	1	Breeding*	2-rowed
B3887	Germany	GER	1	Breeding*	2-rowed
B3918	Germany	GER	1	Breeding*	2-rowed
B4032	Germany	GER	1	Breeding*	2-rowed
B4057	Germany	GER	1	Breeding*	2-rowed
B4167	Germany	GER	1	Breeding*	2-rowed
B4338	Germany	GER	1	Breeding*	2-rowed

B4339	Germany	GER	1	Breeding*	2-rowed
B4403	Germany	GER	1	Breeding*	2-rowed
Barke	Germany	GER	1	Cultivar	2-rowed
Beatrix	Germany	GER	1	Cultivar	2-rowed
Grace	Germany	GER	1	Cultivar	2-rowed
Marthe	Germany	GER	1	Cultivar	2-rowed
Optic	Germany	GER	1	Cultivar	2-rowed
Quench	Germany	GER	1	Cultivar	2-rowed
S008	Germany	GER	1	Breeding*	2-rowed
S036	Germany	GER	1	Breeding*	2-rowed
S040	Germany	GER	1	Breeding*	2-rowed
S054	Germany	GER	1	Breeding*	2-rowed
S095	Germany	GER	1	Breeding*	2-rowed
S101	Germany	GER	1	Breeding*	2-rowed
S112	Germany	GER	1	Breeding*	2-rowed
S118	Germany	GER	1	Breeding*	2-rowed
S120	Germany	GER	1	Breeding*	2-rowed
S140	Germany	GER	1	Breeding*	2-rowed
Scarlett	Germany	GER	1	Cultivar	2-rowed
Triumph	Czech Republic	GER	1	Cultivar	2-rowed
Acsad	Jordan	MED	2	Landrace	2-rowed[§]
ARKE129	Syria	MED	2	Landrace	2-rowed[§]
ARKE130	Syria	MED	2	Landrace	2-rowed[§]
ARKE145	Syria	MED	2	Landrace	2-rowed[§]
ARKE160	Syria	MED	2	Landrace	2-rowed[§]
ARKE182	Syria	MED	2	Landrace	2-rowed[§]
ARKE26	Syria	MED	2	Landrace	2-rowed[§]
ARKE31	Syria	MED	2	Landrace	2-rowed[§]
Arta	Syria	MED	2	Landrace	2-rowed[§]
G400	Egypt	MED	2	Landrace	2-rowed[§]
ER/Apm	North Africa	MED	2	Landrace	2-rowed[§]
Flagship	Australia	MED	2	Cultivar	2-rowed[§]
HID44	Iran	MED	2	Landrace	2-rowed[§]
Keel	Australia	MED	2	Cultivar	2-rowed[§]
LR521	Ethiopia	MED	2	Landrace	6-rowed[§]

MBR1012	Serbia	MED	2	Landrace	2-rowed
Mutah	Jordan	MED	2	Landrace	2-rowed§
RB_119	Egypt	MED	2	Landrace	2-rowed§
RB_162	Iran	MED	2	Landrace	2-rowed§
RB_190	Syria	MED	2	Landrace	2-rowed§
RB_196	Marocco	MED	2	Landrace	2-rowed§
RB_231	Jordan	MED	2	Landrace	2-rowed§
RB_233	Iran	MED	2	Landrace	2-rowed§
RB_242	Tunisia	MED	2	Landrace	2-rowed§
RB_269	Iraq	MED	2	Landrace	2-rowed§
RB_280	Iran	MED	2	Landrace	2-rowed§
RB_86	Algeria	MED	2	Landrace	2-rowed§
RB_88	Pakistan	MED	2	Landrace	2-rowed§
RB_93	Algeria	MED	2	Landrace	2-rowed§
Rum	Jordan	MED	2	Landrace	6-rowed§
Tadmor	Syria	MED	2	Landrace	2-rowed§
Yarmouk	Jordan	MED	2	Landrace	2-rowed§
Bowman	USA	MED	2	Cultivar	2-rowed§
Morex	USA	MED	2	Cultivar	6-rowed
Streptoe	USA	MED	2	Cultivar	6-rowed

Supplementary Figures

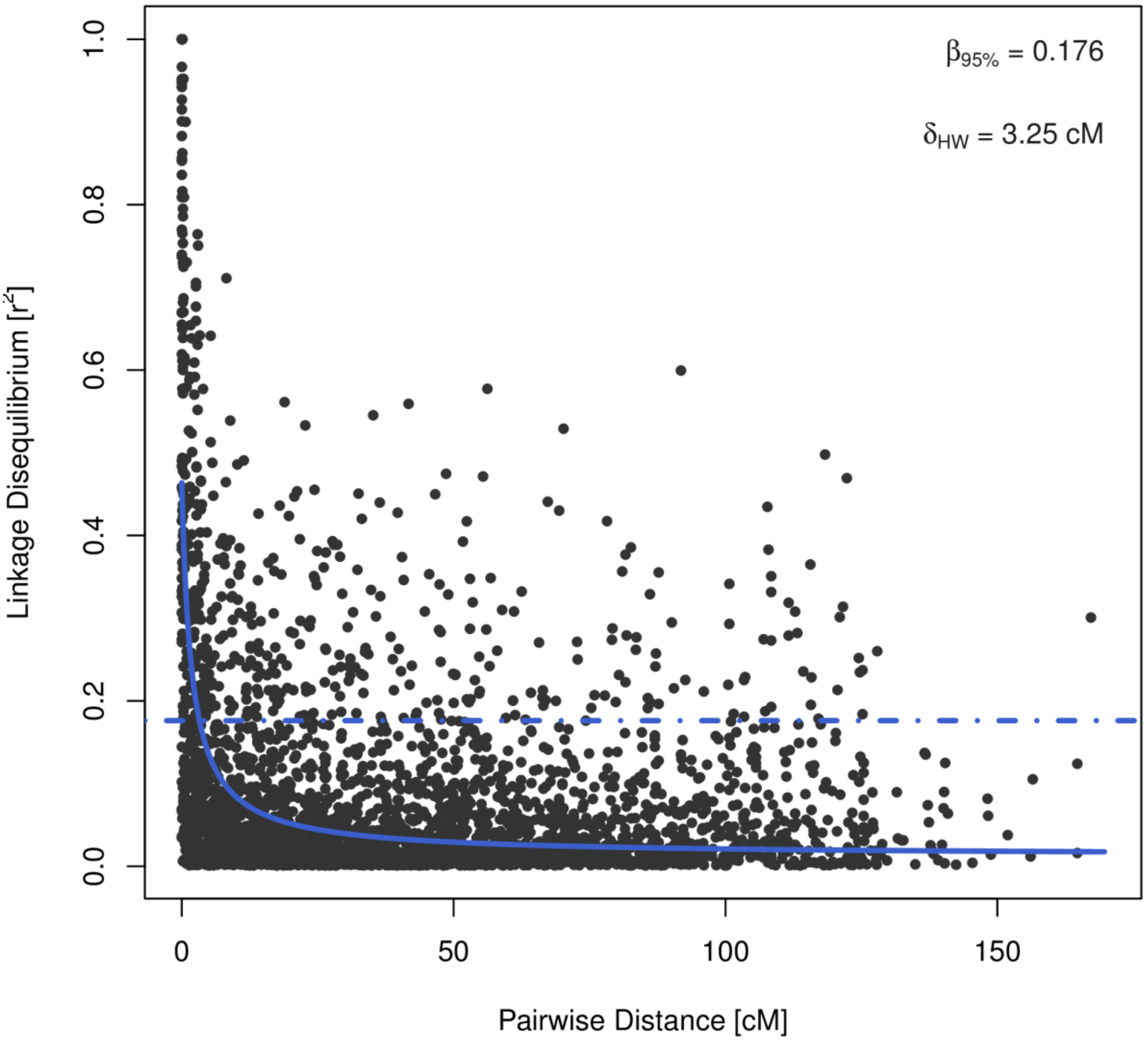

Figure S1. Linkage disequilibrium in the investigated barley germplasm collection. Decay of LD (r^2) with distance between pairs of SNPs. The value of r^2 = 0.176 was reached at approximately 3.25 cM (δ_{HW}) at a confidence level of 95%.

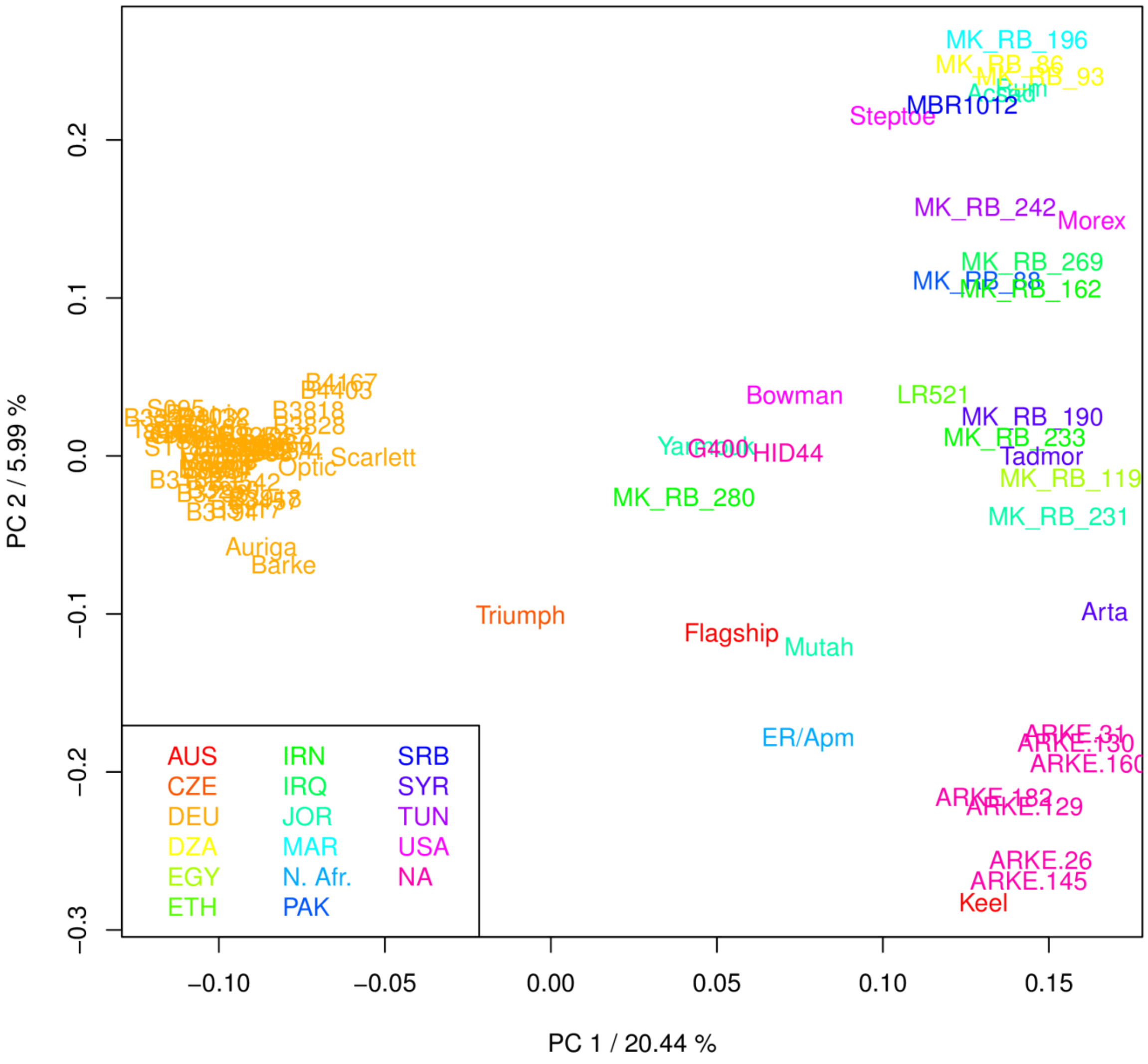

Figure S2. Principal component analysis of the genetic structure of the investigated barley collection.

The genetic diversity of the investigated barley panel was analysed by principal component analysis (PCA) of 2596 informative SNP markers of the Illumina 9K SNP array from all investigated 81 genotypes.

For geographic origin of the individual lines, please see the inset. AUS – Australia, CZE – Czech Republic, DEU – Germany, DZA – Algeria, EGY – Egypt, ETH – Ethiopia, IRN – Iran, IRQ – Iraq, JOR – Jordan, MAR – Morocco, N. Afr. – other North African countries, PAK – Pakistan, SRB – Serbia, SYR – Syria, TUN – Tunisia, USA . United States, NA – Miscellaneous.

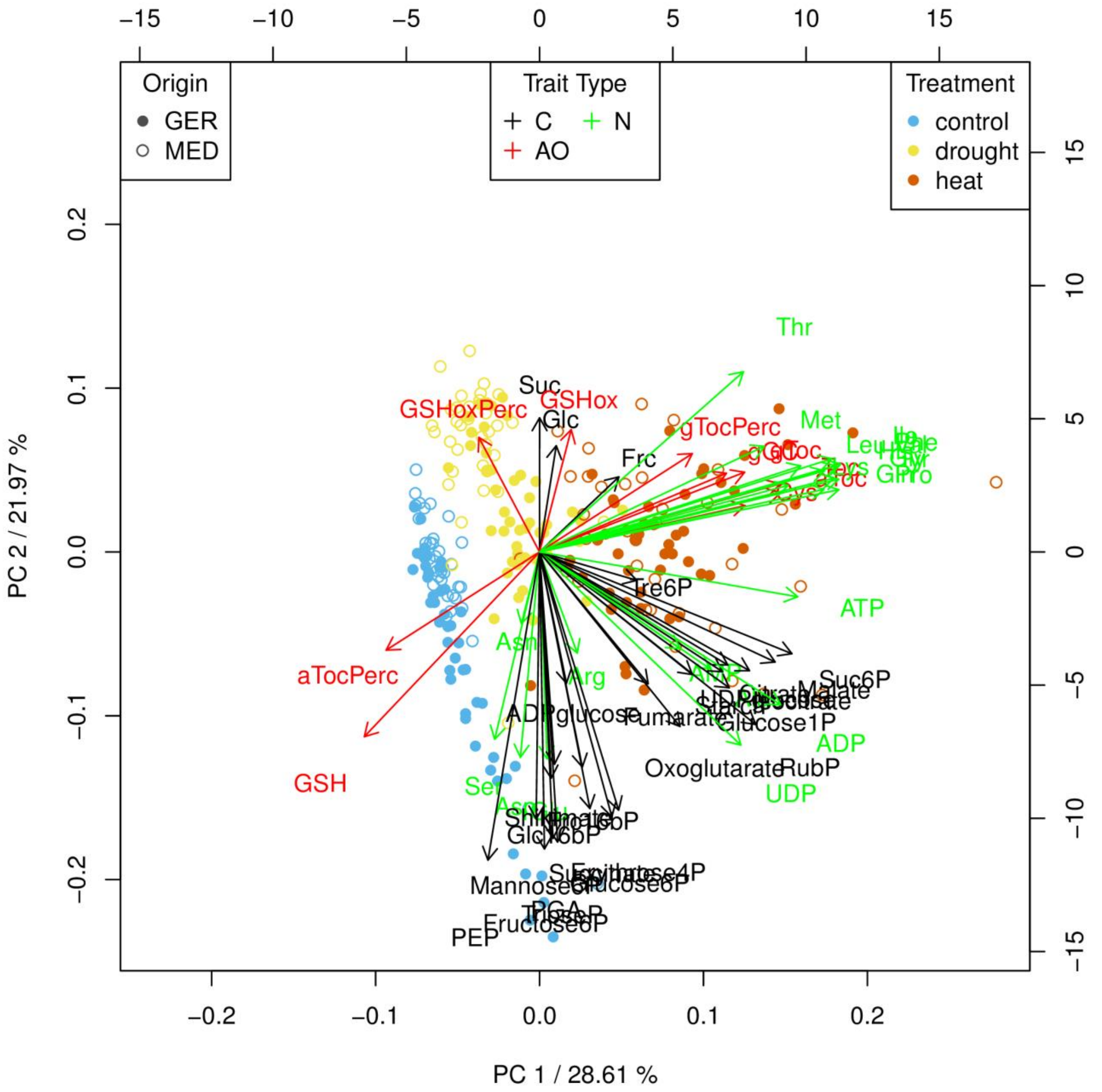

Figure S3. PCA based on metabolite data from all three growth regimes.

A, Analysis of the metabolite dataset from 46 GER and 35 MED genotypes excluding agronomic and physiological parameters from all three conditions (control, drought, combined heat and drought).

Metabolite data of 4 replicate flag leaf pools per genotype and treatment sampled at 3 days after stress establishment in one of three replicate experiments were used. All other data were taken from three independent experiments, with 4 replicates per genotype and treatment, each: physiological parameters (PSII intactness (Fv/Fm value), RWC and leaf temperature) were quantified between 3 and 10 days after stress as described in the materials and methods section, while agronomic data were determined at final harvest.

Lower abscissa and left ordinate show PC scores of PC1 and PC2, respectively, while upper abscissa and right ordinate show loading scores for PC1 and PC2, respectively. Circles

indicate the position of the individual genotypes in the plotted two-dimensional space of principal component 1 (PC1) and PC2. GER - filled circles, MED - open circles, control - blue symbols, drought – yellow symbols, combined heat and drought – red symbols. Arrows indicate the loadings of metabolite, physiological and agronomic traits. Carbon metabolites – black arrows, nitrogen metabolites – green arrows, antioxidants – red arrows.

Amino acids and nucleotides are abbreviated according to standard three letter code, Oxoglutarate (α-ketoglutarate), aToc (α-tocopherol), aTocPerc (% α-tocopherol), Erythrose4P (erythrose-4-phosphate), F16bP (fructose-1,6-bisphosphate), Fructose6P (fructose-6-phosphate), Frc (fructose), gGC (γ-glutamyl cysteine), gToc (γ-tocopherol), gTocPerc (% γ-tocopherol), G16bP (glucose-1,6-bisphosphate), Glucose1P (glucose-1-phosphate), Glucose6P (glucose-6-phosphate), Glc (glucose), GSH (glutathione), GSHoxPerc (% glutathione oxidized), Mannose6P (mannose-6-phosphate), PEP (phospho*enol* pyruvate), PGA (3-phosphoglycerate), RubP (ribulose-1,5-bisphosphate), Shik (shikimate), Sucrose6P (sucrose-6-phosphate), Suc (sucrose), Toc (total Tocopherol), TriP (Triose-phosphates), Tre6P (trehalose-6-phosphate), UDPglc (UDP-glucose).

LWC (relative leaf water content), LT1 (leaf temperature), YBM (total biomass), YST (straw biomass), YGR (grain yield).

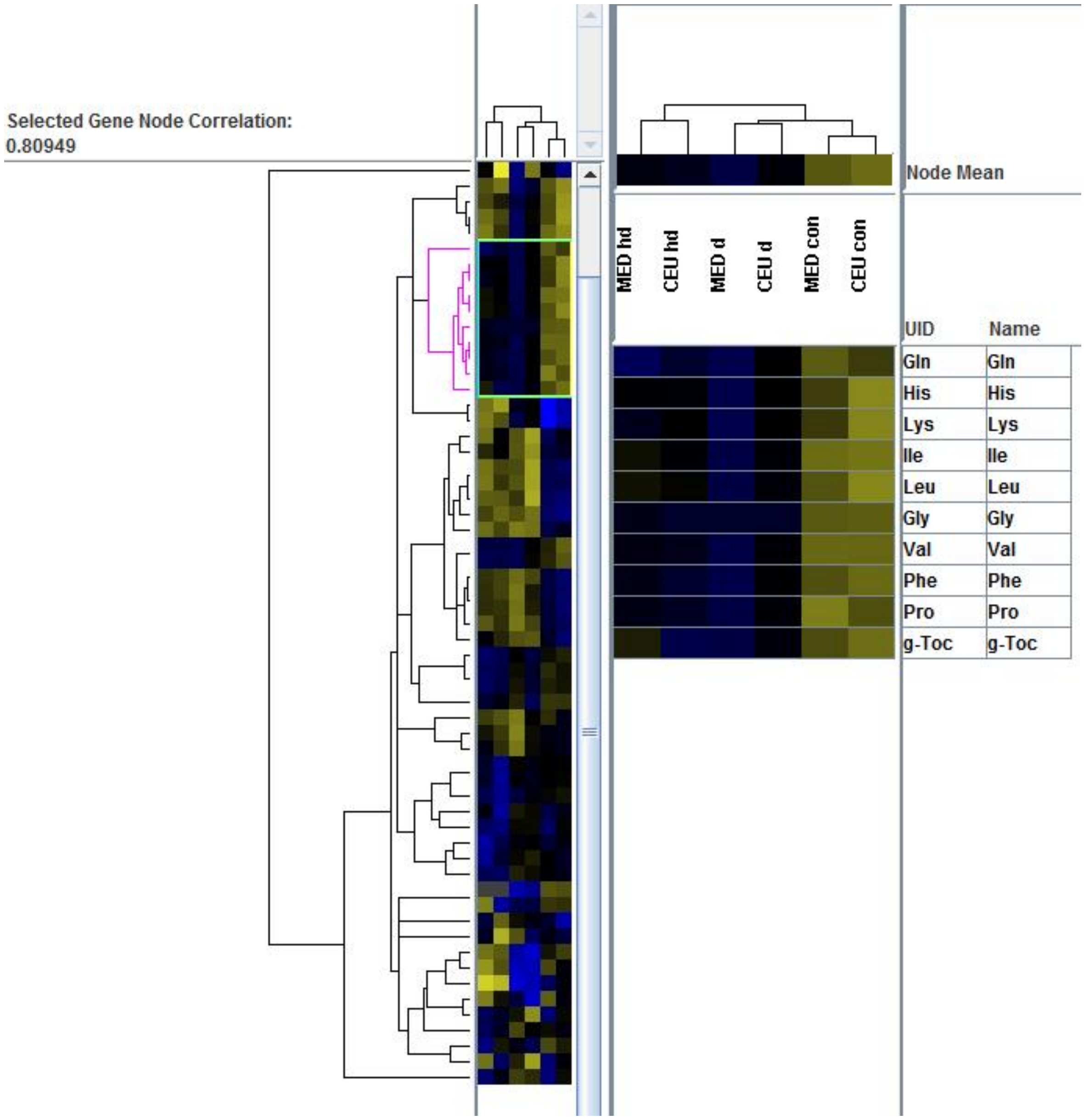

Figure S4A. Hierarchcical cluster analysis (HCA) of metabolite data with highlighted subclades.

A, HCA, in which a clade of metabolites is highlighted (by a green box on the left side of the figure) that accumulate stronger in combined stress compared to drought stress. The boxed clade is magnified on the right side of the figure including the names of the metabolites.

Mean values for metabolite data were calculated separately for all 46 GER (CEU) and all 35 MED genotypes prior to HCA. False color scale designates blue fields as below median and yellow fields as above median. con – control, d – drought, hd – combined heat and drought. A zoomed image of the highlighted clade of metabolites is shown on the right hand side and includes the names of the individual metabolites of the labeled clade.

Amino acids and nucleotides are abbreviated according to standard three letter code, g-Toc (γ-tocopherol).

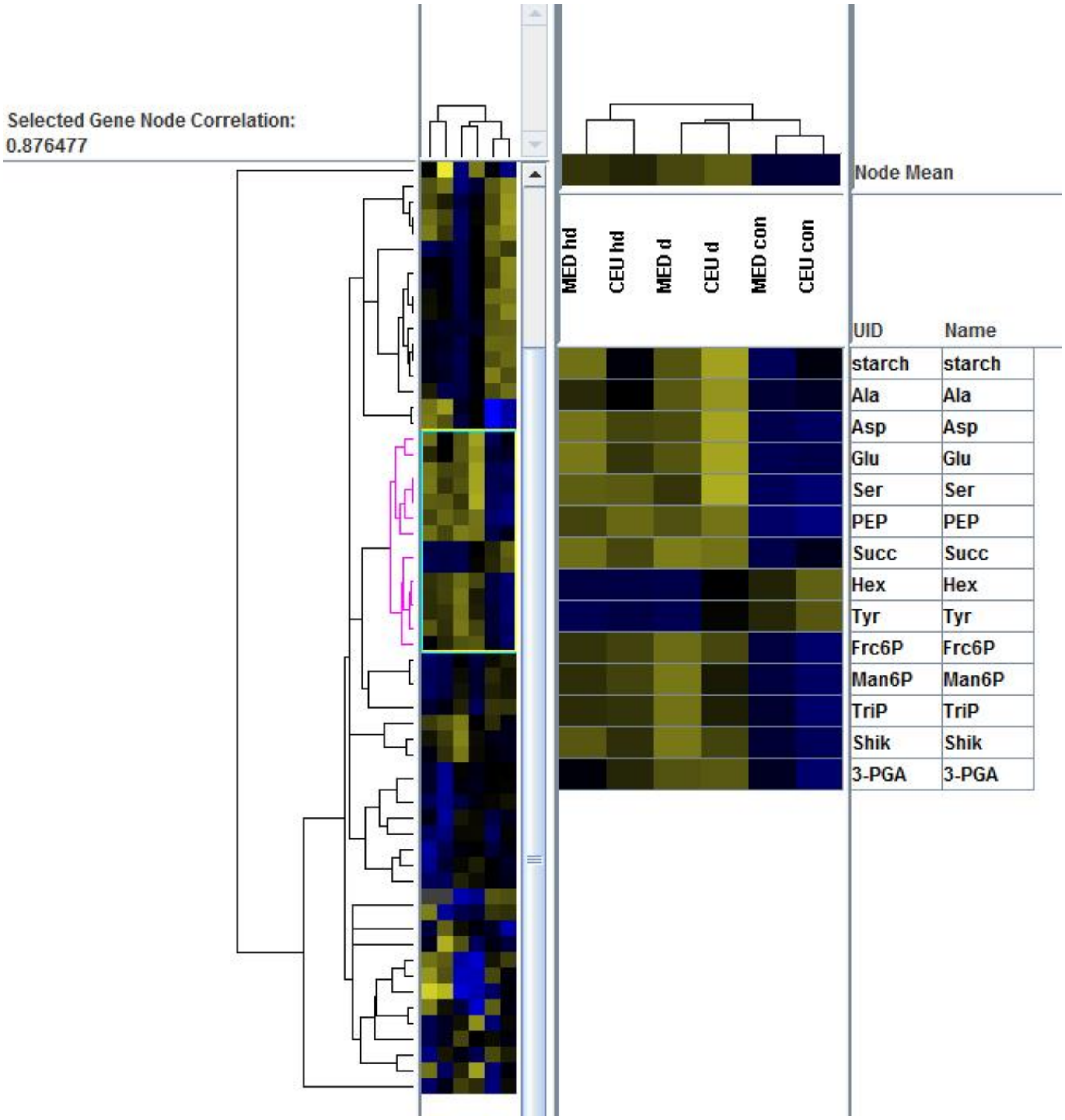

Figure S4B. Hierarchcical cluster analysis (HCA) of metabolite data with highlighted subclades.

B, HCA, in which a clade of metabolites is highlighted (by a green box on the left side of the figure) that accumulates less in drought compared to the other two conditions. The boxed clade is magnified on the right side of the figure including the names of the metabolites.

Mean values for metabolite data were calculated separately for all 46 GER (CEU) and all 35 MED genotypes prior to HCA. False color scale designates blue fields as below median and yellow fields as above median. con – control, d – drought, hd – combined heat and drought. A zoomed image of the highlighted clade of metabolites is shown on the right hand side and includes the names of the individual metabolites of the labeled clade.

Amino acids and nucleotides are abbreviated according to standard three letter code, Frc6P (fructose-6-phosphate), Frc (fructose), Glc (glucose), Hex (Hexoses, i.e. Glc + Frc), Man6P (mannose-6-phosphate), PEP (phospho*enol* pyruvate), 3-PGA (3-phosphoglycerate), Shik (shikimate), Succ (succinate), TriP (Triose-phosphates),

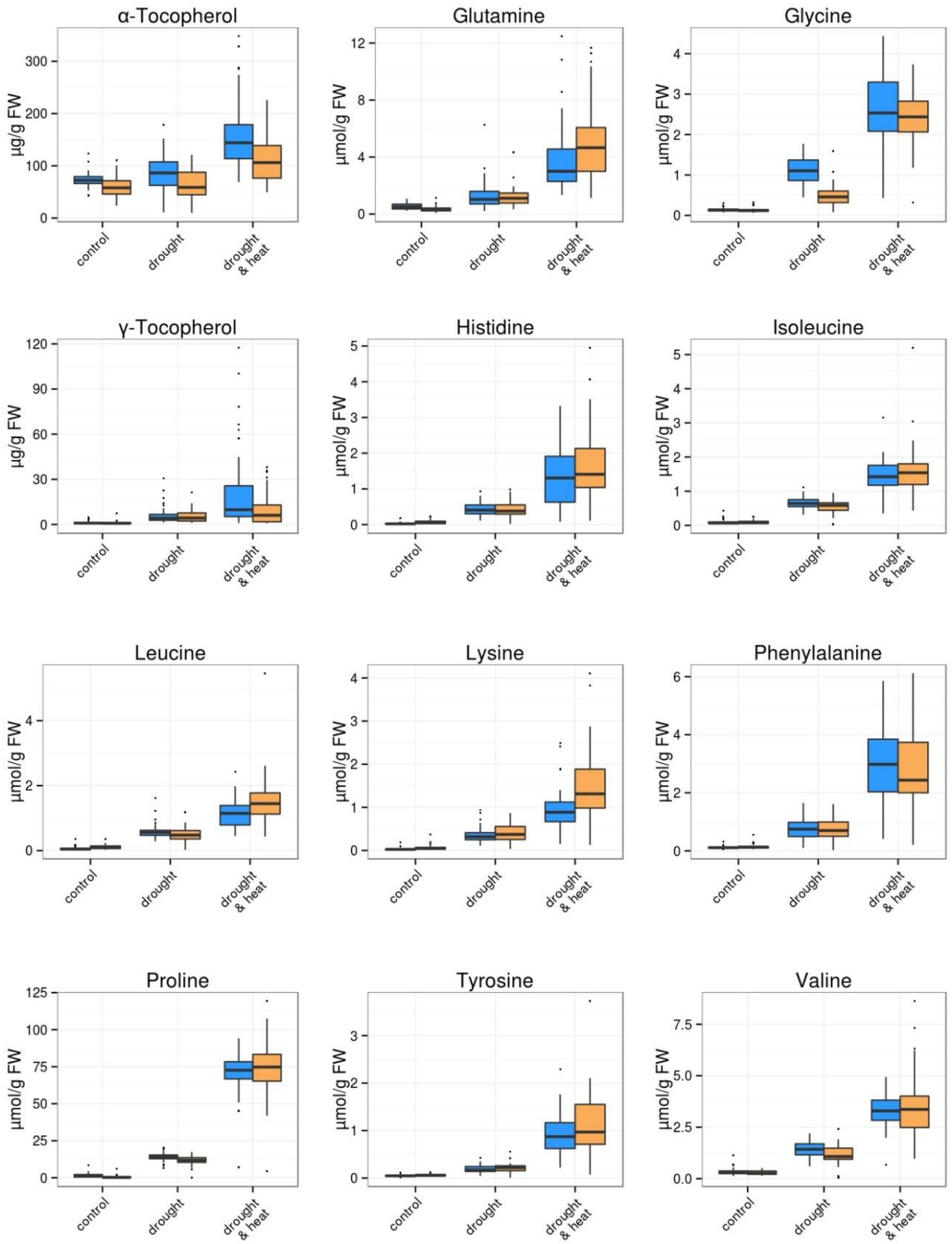

Figure S5. Metabolites that accumulate stronger in flag leaves at 3 days after stress establishment under combined than in drought stress.

Data of 4 replicates per genotype and treatment from 46 GER lines (blue bars) and 35 MED lines (orange bars) were grouped for analysis and are shown from control (left), drought stress (middle) and combined heat and drought (right). Results of the ANOVA are provided in Table S2. In the box plots, the median ± upper and lower quartile is indicated as boxes.

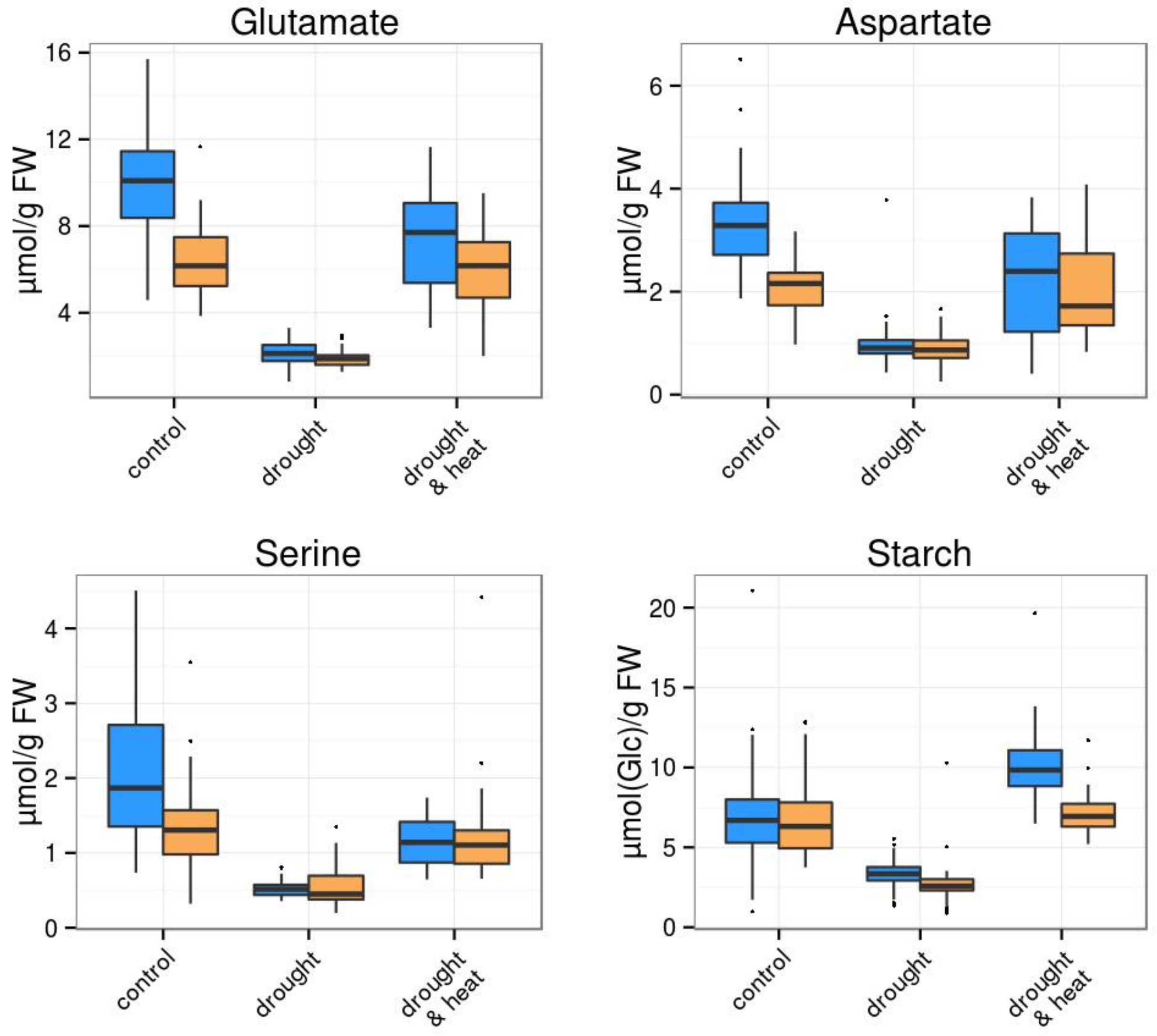

Figure S6. Metabolites with diminished steady state levels in drought stressed flag leaves at 3 days after stress establishment.

Data of 4 replicates per genotype and treatment from 46 GER lines (blue bars) and 35 MED lines (orange bars) were grouped for analysis and are shown from control (left), drought stress (middle) and combined heat and drought (right). Results of the ANOVA are provided in Table S2. In the box plots, the median ± upper and lower quartile is indicated as boxes.

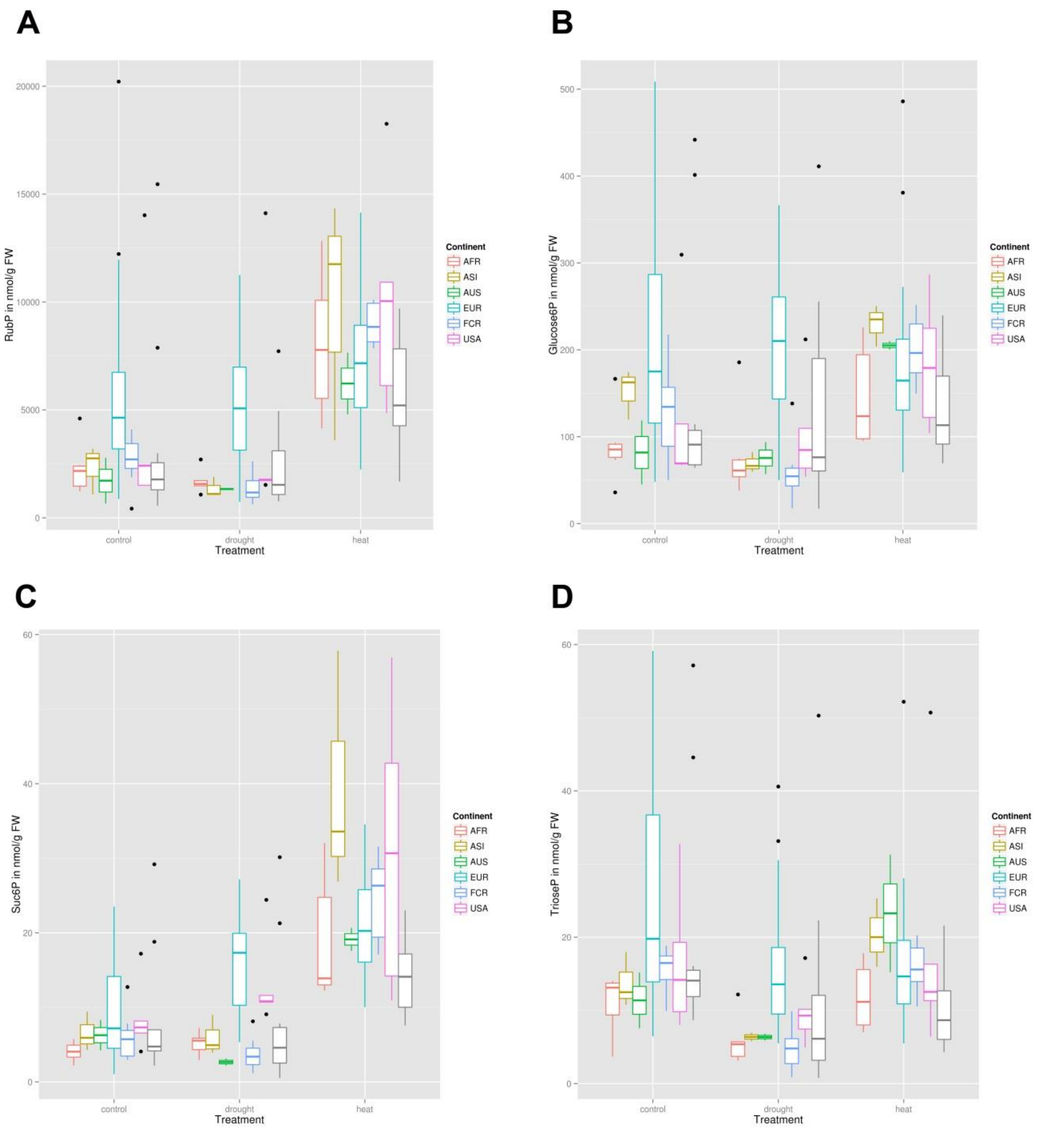

Figure S7. Steady state contents of phosphorylated intermediates in flag leaves at 3 days after stress establishment by geographic origin.

A, Ribulose-1,5-bisphosphate; B, Glucose-6-phosphate; C, Sucrose-6-phosphate; D, Triosephosphates.

Data of 4 replicates per genotype and treatment from 8 genotypes of african origin (AFR, orange bars), 5 genotypes of asian origin (ASI, ochre bars), 2 australian cultivars (AUS, green bars), 46 german elite lines (EUR, turquoise bars), 9 genotypes from the fertile crescent (FCR, blue bars), 3 US lines (USA, pink bars) as well as from all genotypes (grey bars) were grouped for analysis and are shown from control (left), drought stress (middle) and combined heat and drought (right). In the box plots, the median ± upper and lower quartile is indicated as boxes.

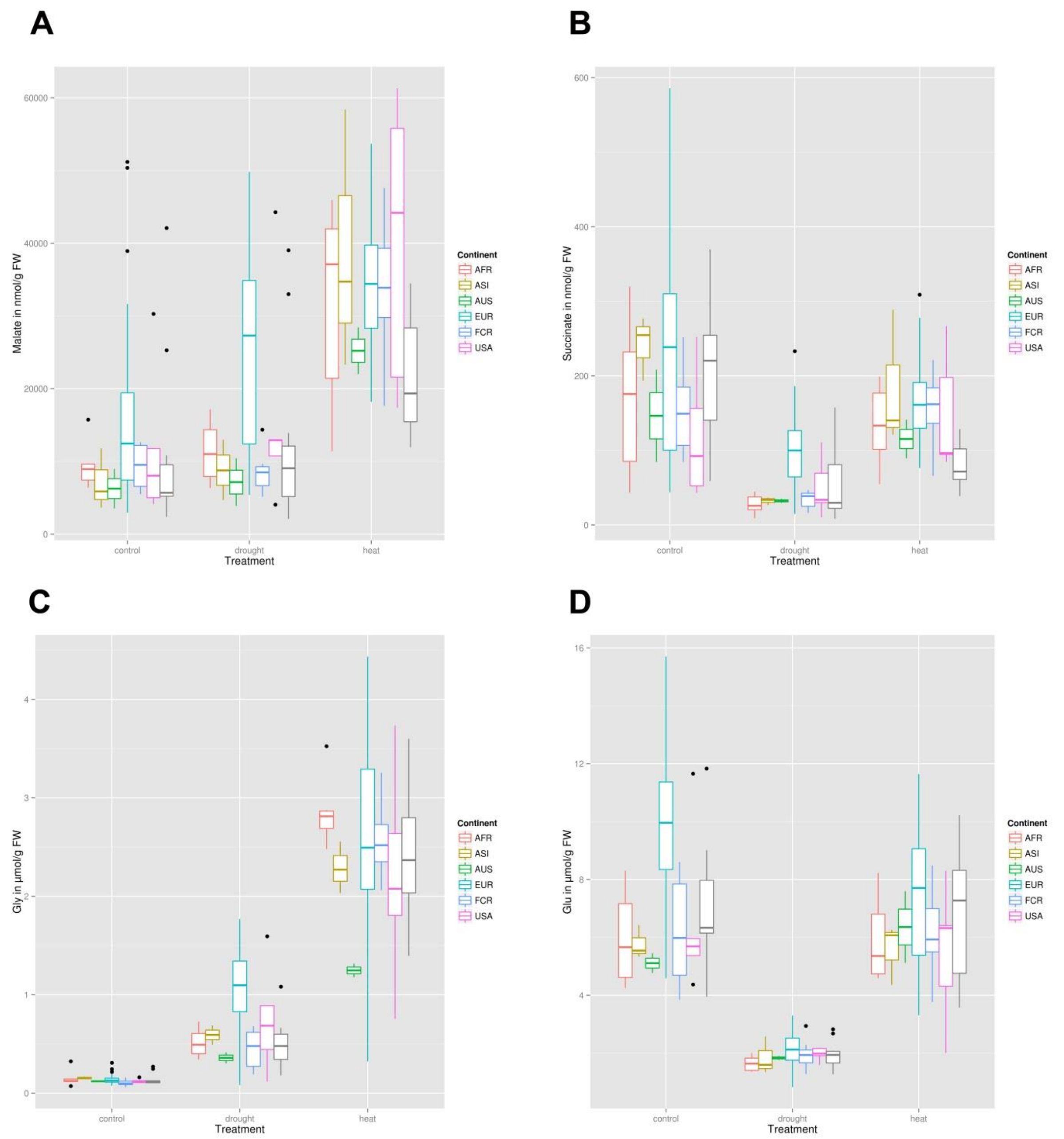

Figure S8. Steady state contents of carboxylates and amino acids in flag leaves at 3 days after stress establishment by geographic origin.

A, Malate; B, Succinate; C, Glycine; D, Glutamate.

Data of 4 replicates per genotype and treatment from 8 genotypes of african origin (AFR, orange bars), 5 genotypes of asian origin (ASI, ochre bars), 2 australian cultivars (AUS, green bars), 46 german elite lines (EUR, turquoise bars), 9 genotypes from the fertile crescent (FCR, blue bars), 3 US lines (USA, pink bars) as well as from all genotypes (grey bars) were grouped for analysis and are shown from control (left), drought stress (middle) and combined heat and drought (right). In the box plots, the median ± upper and lower quartile is indicated as boxes.

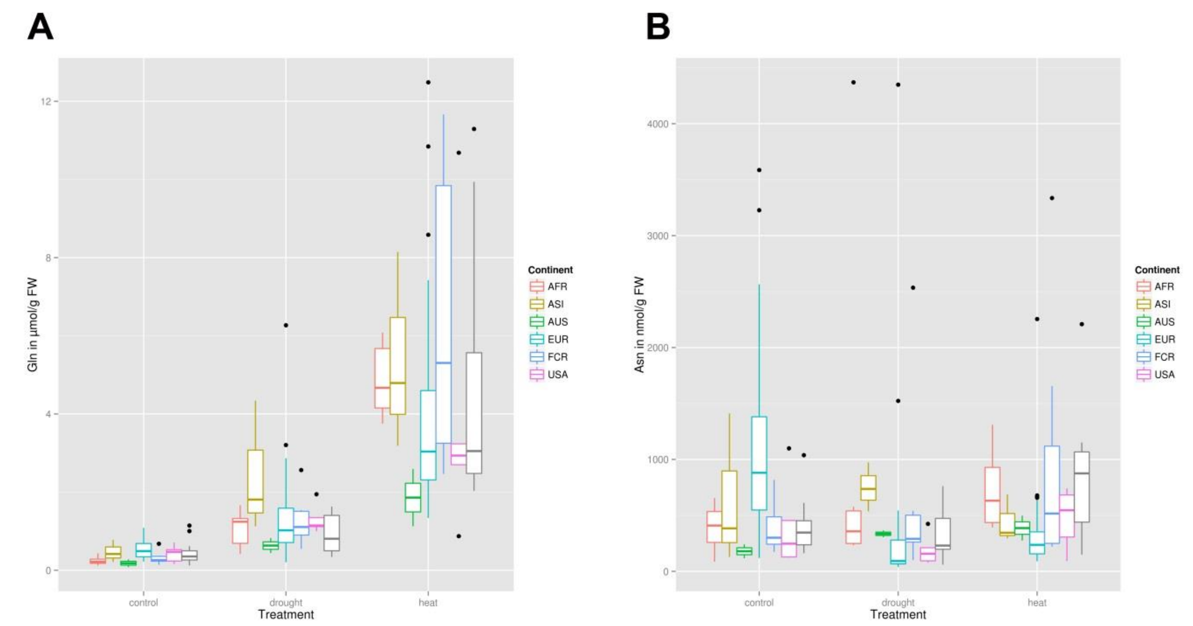

Figure S9. Steady state contents of glutamine and asparagine in flag leaves at 3 days after stress establishment by geographic origin.

A, Glutamine; B, Asparagine

Data of 4 replicates per genotype and treatment from 8 genotypes of african origin (AFR, orange bars), 5 genotypes of asian origin (ASI, ochre bars), 2 australian cultivars (AUS, green bars), 46 german elite lines (EUR, turquoise bars), 9 genotypes from the fertile crescent (FCR, blue bars), 3 US lines (USA, pink bars) as well as from all genotypes (grey bars) were grouped for analysis and are shown from control (left), drought stress (middle) and combined heat and drought (right). In the box plots, the median ± upper and lower quartile is indicated as boxes.

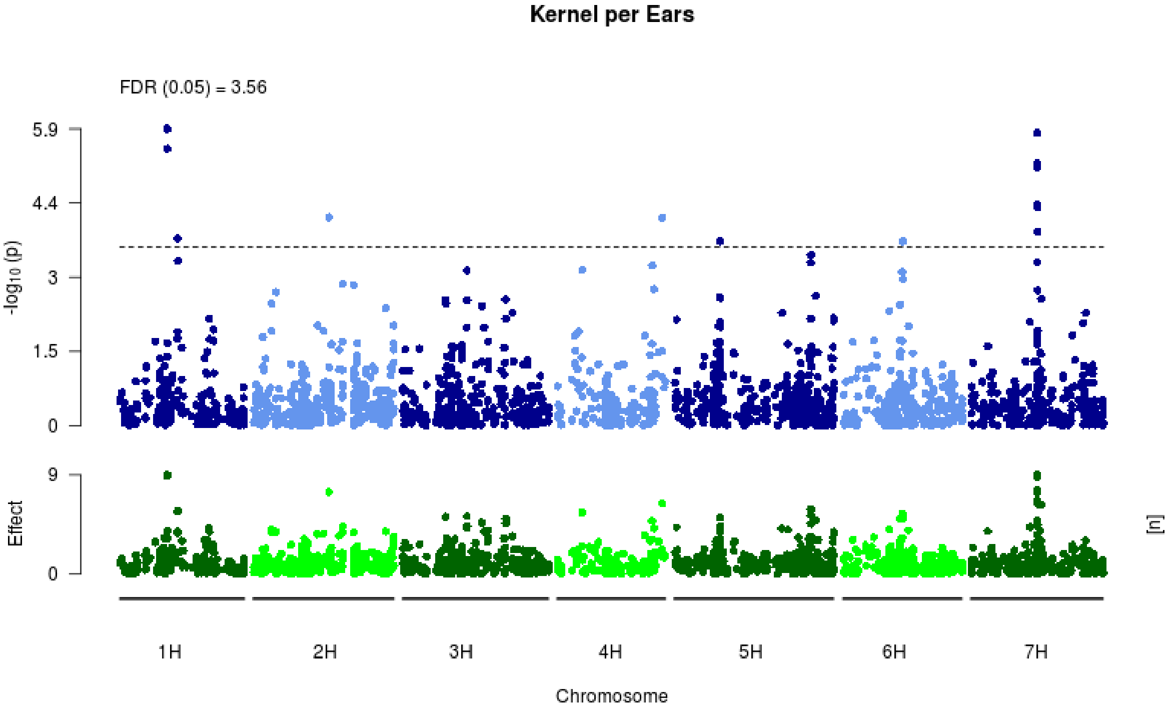

Figure S10. Manhattan plots showing the genome wide association of morphological traits with SNP markers.

Manhattan plots for the association analysis of the morphological trait Kernel Per Ears from a GWAS over all treatments (as fixed effect). Each dot along the x-axis represents the negative logarithmic p-value (blue) and effect size (green) of a single SNP marker.
